# $\pi _1$ of Miranda moduli spaces of elliptic surfaces

**DOI:** 10.1007/s40574-021-00297-2

**Published:** 2021-06-28

**Authors:** Michael Lönne

**Affiliations:** Mathematisches Institut der Universität Bayreuth, Universitätsstr. 30, 95447 Bayreuth, Germany

**Keywords:** elliptic surfaces, moduli spaces, orbifold fundamental group, braid monodromy, discriminant knot group

## Abstract

We give finite presentations for the fundamental group of moduli spaces due to Miranda of smooth Weierstrass curves over ${\mathbf {P}}^1$ which extend the classical result for elliptic curves to the relative situation over the projective line. We thus get natural generalisations of $SL_2{{\mathbb {Z}}}$ presented in terms of $$\Bigg (\begin{array}{ll} 1&{}1\\ 0&{}1\end{array} \Bigg )$$, $$\Bigg (\begin{array}{ll} 1&{}0\\ {-1}&{}1\end{array} \Bigg )$$ on one hand and the first examples of fundamental groups of moduli stacks of elliptic surfaces on the other.

Our approach exploits the natural ${\mathbb {Z}}_2$-action on Weierstrass curves and the identification of ${\mathbb {Z}}_2$-fixed loci with smooth hypersurfaces in an appropriate linear system on a projective line bundle over ${{\mathbf {P}}}^1$. The fundamental group of the corresponding discriminant complement can be presented in terms of finitely many generators and relations using methods in the Zariski tradition.

## Introduction

Our primary objects are curves on the ruled surface $X_{d}={\mathbf {P}}\left( {\mathcal {O}}_{{\mathbf {P}}^1}(d)\oplus {\mathcal {O}}_{{\mathbf {P}}^1}\right)$ in the linear system $|3\sigma _0|$, where $\sigma _0$ denotes the divisor on $X_{d}$ defined by the zero section of ${\mathcal {O}}_{{\mathbf {P}}^1}(d)$. They form a universal hypersurface ${\mathcal {H}}_{d}$ in $X_{d}\times {\mathbf {P}}V_{d}$, $V_{d}=\Gamma (X_{d},{\mathcal {O}}_{X_{d}}(3\sigma _0))$.

Upon a choice of homogeneous coordinates $y,y_0$ on a fibre and $x_0,x_1$ on the base, $V_{d}$ is identified with the polynomials of ${\mathbb {C}}[y_0,y,x_0,x_1]$ which have degree 3 in the variables $y_0,y$ and weighted degree 3*d* in the variables $y,x_i$ of weights *d* and 1 respectively.

On $V_{d}$ we introduce coordinates $u^{\circ }, u''_{\nu }, u'_{\nu }, u_{\nu }$ with respect to the monomial basis such that the tautological hypersurface ${\mathcal {H}}_{d}$ is given by the vanishing of

1 $$\begin{aligned} u^{\circ } y^{3} \,+\,\sum _{\nu +\mu =d} u''_\nu y_0y^2 x_0^\nu x^\mu \,+\,\sum _{\nu +\mu =2d} u'_\nu y_0^2y x_0^\nu x^\mu \,+\,\sum _{\nu +\mu =3d} u_\nu y_0^3 x_0^\nu x^\mu . \end{aligned}$$

Its projection to the factor ${\mathbf {P}}V_{d}$ has singular values precisely along the discriminant

$$\begin{aligned} {\mathcal {D}}_{d}= & {} \big \{ u\in {{\mathbf {P}}} V_{d}\, \big | \, \text {the fibre } {\mathcal {H}}|_u \text { is singular} \big \} \end{aligned}$$

which is the union of the hyperplane $\{u^{\circ }=0\}$ and the projective dual of the weighted projective plane ${\mathbf {P}}^{2}_{d,1,1}$ given as the image of $X_{d}$ under the projective morphism defined by the base point free linear system $|3\sigma _0|$.

The problem we want to address in the first stage is to give a geometrically distinguished finite presentation of the fundamental group of the complement ${\mathcal {U}}_{d}$ of ${\mathcal {D}}_{d}$. It may be viewed as a special instance of the vastly open problem posed by Dolgachev and Libgober, [1], to determine the fundamental group of the discriminant complement of any (complete) linear system.

The first result of that kind, actually, is due to Zariski who considered the complete linear systems on ${\mathbf {P}}^1$. It already exhibits some typical features; most relations impose a commutation or a braid relation between elements or even generators and there is a relation due to the action of ${\mathbb {C}}^*$.

### Theorem 1

(Zariski [13] and Fadell, van Buskirk [3]) The fundamental group $\pi _1({\mathcal {U}}_{{\mathbf {P}}^1,l})$ of the discriminant complement associated to the complete linear system of degree *l* on ${\mathbf {P}}^1$ is finitely presented by generators $\sigma _1,...,\sigma _{l-1}$ and relations

(i) $$\sigma _{i}\sigma _{j}=\sigma _{j}\sigma _{i}$$, if $$|i-j|{\ge 2}$$, $$1\le i,j<l$$,
(ii) $$\sigma _{i}\sigma _{i+1}\sigma _{i}=\sigma _{i+1}\sigma _{i}\sigma _{i+1}$$, if $$\,1\le i<l-1$$,
(iii) $$\sigma _{1}\dots \sigma _{l-2}\sigma _{l-1}\sigma _{l-1}\sigma _{l-2}\dots \sigma _1=1$$.

We have previously extended this result to complete linear systems on projective spaces, [6], and we provide now a series of examples of linear systems on ruled surfaces:

### Theorem 2

**Fig. 1:**
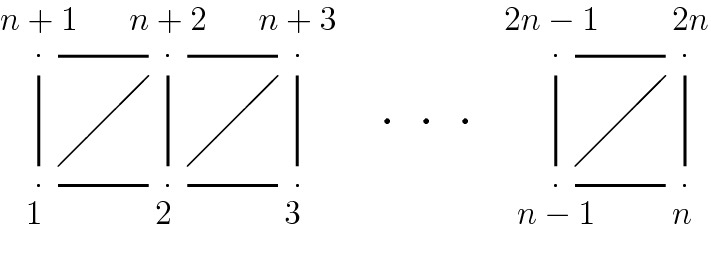
The graph $$\Gamma _n$$

Suppose ${\mathcal {U}}_{d}$ is the discriminant complement in the linear system $|3 \sigma _0|$ on the ruled surface ${\mathbf {P}}({\mathcal {O}}(d)\oplus {\mathcal {O}})$. Then $\pi _1({\mathcal {U}}_{d})$ is generated by elements

$$\begin{aligned} {t_{1}}, \dots , {t_{2n}}, \quad n=3d-1 \end{aligned}$$

with the following complete set of relations, (i)–(iii) provided in terms of the edges $E_n$ of the graph $\Gamma _n$ (Fig. 1):

(i) for all $$(i,j)\not \in E_{d}$$
  $$\begin{aligned} t_{i} t_{j}= t_{j} t_{i} \end{aligned}$$
(ii) for all $(i,j)\in E_{d}$
  $$\begin{aligned} t_{i} t_{j} t_{i}= t_{j} t_{i} t_{j} \end{aligned}$$
(iii) for $$(i,j),(i,k),(j,k)\in E_{d}$$, $$i<j<k$$
  $$\begin{aligned} t_{i} t_{j} t_{k} t_{i}= t_{j} t_{k} t_{i} t_{j} \end{aligned}$$
(iv) for $j=n$ and $j=2n$
  $$\begin{aligned} \bigg ( t_{j}{^{^{-1}}} \Big ( t_{2n} t_{2n-1} \cdots t_{2} t_{1} \Big )\bigg )^{{3d}-1} \,=\quad \bigg (\Big ( t_{2n} t_{2n-1} \cdots t_{2} t_{1} \Big ) t_j^{^{-1}}\bigg )^{{3d}-1} \end{aligned}$$

Still our principle aim is to find similar presentations for the fundamental groups of moduli spaces of elliptic surfaces. Recall that Miranda [10] constructed coarse moduli spaces of regular elliptic surfaces with a section as a *G.I.T.*-quotient of suitably fixed Weierstrass data.

Indeed such datum determines an elliptic surface given as a hypersurface by a Weierstrass equation in the ${\mathbf {P}}^2$-bundles $Y_{d}= {\mathbf {P}}\left( {\mathcal {O}}_{{\mathbf {P}}^1}\oplus {\mathcal {O}}_{{\mathbf {P}}^1}(d) \oplus {\mathcal {O}}_{{\mathbf {P}}^1}(3d/2)\right)$, where *d* is even. We introduce homogeneous coordinates $y_0,y=y_1,y_2$ on the fibres and $x_0,x_1$ on the base. A Weierstrass fibration is then defined to be given by an equation of the form

2 $$\begin{aligned} y_0y_2^2= y^34 \,+\,\sum _{\nu +\mu =2d} u'_\nu y_0^2y x_0^\nu x^\mu \,+\,\sum _{\nu +\mu =3d} u_\nu y_0^3 x_0^\nu x^\mu , \end{aligned}$$

where the coefficients $u'_\nu , u_\nu$ are coordinates of a vector subspace $V_{n,d}'$ of $V_{n,d}$ spanned by the monomials $x_0^\nu x^\mu ,\nu +\mu =2d, 3d$.

The same equation also defines the associated tautological Weierstrass hypersurface ${\mathcal {W}}_{d}$ in $Y_{n,d}\times V_{n,d}'$. Its projection to $V_{n,d}'$ has singular values along the discriminant

$$\begin{aligned} {\mathcal {D}}_{n,d}' \quad = \quad {\mathcal {D}}_{n,d}\, \cap \, V_{n,d}' \end{aligned}$$

where $V_{n,d}'$ is embedded into ${\mathbf {P}}V_{n,d}$ as the affine part (w.r.t. the hyperplane $u^{\circ }=0$) of the projective subspace generated by $V_{d}'$ and $y^3$, the monomial with coefficient $u^{\circ }$.

That last property is easily checked on equations and reflects the following fact. A Weierstrass fibration is a double cover of a smooth toric surface and therefore smooth if the ${\mathbb {Z}}_2$-fixed locus is. Its fixed part off the hypersurface $y_2=0$ is always smooth; hence smoothness is equivalent to smoothness of the restriction to $y_2=0$ which yields precisely the smoothness condition considered in the first part.

The complement ${\mathcal {U}}_{d}'$ of ${\mathcal {D}}_{d}'$ in $V_{d}'$ is the base of a versal family of smooth Weierstrass fibrations. Remember that Miranda constructs his moduli space to coarsely represent all smooth regular elliptic surfaces of Euler number 6*d* with a section. Here instead, we discard the singular Weierstrass fibrations which are associated to smooth elliptic surfaces with reducible fibres.

Accordingly we obtain an open part ${\mathcal {M}}_{d}$ of the Miranda moduli space as the quotient of $V_{d}'$ by the group of equivalences, which is given as the direct product of the group of linear projective transformations of the base and a torus ${\mathbb {C}}^*$ acting on the coordinates $u_\nu ,u'_\nu$ see Sect. 5. We will show $\pi _1({\mathcal {U}}_{d})\cong \pi _1({\mathcal {U}}_{d}')$ and derive the orbifold fundamental group of ${\mathcal {M}}_{d}$ from a homotopy exact sequence.

In this way we are able to generalise to base dimension one the old result giving the orbifold fundamental group of the moduli space of elliptic curves, which is naturally the moduli space of smooth Weierstrass fibrations over the point.

### Theorem 3

The (orbifold) fundamental group $SL_2{{\mathbb {Z}}}$ of ${\mathcal {M}}_{0}$ is finitely presented as

$$\begin{aligned} \langle \quad \sigma _{1},\sigma _{2} \quad | \quad \sigma _{1} \sigma _{2} \sigma _{1} \quad = \quad \sigma _{2}\sigma _{1}\sigma _{2},\quad (\sigma _{1}\sigma _{2})^{6} = 1\rangle \end{aligned}$$

Of course this natural generalisation relies heavily on our theorem 2:

### Theorem 4

The (orbifold) fundamental group $\pi _1({\mathcal {M}}_{n,d})$, *d* even, is generated by elements $t_i,\,1\le i\le 2n$, $n=3d-1$ in bijection to the vertices of the graph $\Gamma _{n}$, and a complete set of relations is given by $(i)-(iv)$ above and two additional relations

$$\begin{aligned} \mathrm{(v)}\quad \left( t_{n+1} t_{1} \, \dots \, t_{2n}t_{n} \right) ^6 \big ( t_{2n}t_{n} \; \dots \; t_{n+1}t_{1} \big )^6 \quad = \; 1 \; =\quad \big ( t_{2n}t_{n} \; \dots \; t_{n+1}t_{1} \big )^{3d} \end{aligned}$$

In an appendix we will manipulate our presentations and derive the following less involved presentation which uses only relation without inverses of generators.

### Corollary 1

The fundamental group has a presentation with generators $\sigma _1,\dots , \sigma _{2n}$, $n={3d-1}$, and relations:

$$\begin{aligned}&\sigma _i\sigma _j\sigma _i = \sigma _j\sigma _i\sigma _j&|i-j|\le 2\\&\sigma _i\sigma _j=\sigma _j\sigma _i&|i-j|>2\\&\sigma _{i-1}\sigma _{i}\sigma _{i+1}\sigma _{i-1}=\sigma _{i+1}\sigma _{i-1}\sigma _{i} \sigma _{i+1}&1<i<2n\\&(\sigma _1\sigma _2 \dots \sigma _{2n})^{3d}\\&(\sigma _1\sigma _2 \dots \sigma _{2n})^6(\sigma _{2n-1}\sigma _{2n}\; \sigma _{2n-3}\sigma _{2n-2} \;\dots \; \sigma _1 \sigma _2)^6\\&(\sigma _1\sigma _2 \dots \sigma _{n})( \sigma _{2n-1} \sigma _{2n-3} \dots \sigma _{3} \sigma _{1}) = ( \sigma _{2n-1} \sigma _{2n-3} \cdots \sigma _{3}\sigma _{1})(\sigma _1\sigma _2 \cdots \sigma _{n})\\&(\sigma _1\sigma _2 \dots \sigma _{n})( \sigma _{2n} \sigma _{2n-2} \dots \sigma _{4}\sigma _{2}) = ( \sigma _{2n} \sigma _{2n-2} \dots \sigma _{4}\sigma _{2})(\sigma _1\sigma _2 \dots \sigma _{n}) \end{aligned}$$

Let us stress the fact that the relations (i)–(iii) of our presentations have a distinctive flavour since they stem from a different setting: If we consider the Brieskorn-Pham polynomial in the variables *y*, *x*,

$$\begin{aligned} f= y^3\,+\,x^{n+1} \end{aligned}$$

we are naturally led to consider a versal unfolding of the isolated hypersurface singularity it defines. In fact the complement of the discriminant in the unfolding base was shown to have fundamental group generated as in the theorem but with relations (i)–(iii) only, [7], in terms of the graph $\Gamma _n$, which incidentally is a distinguished Dynkin graph associated to the singularity of *f*.

We will explain in detail in Sect. 3 how this result is used in the present paper. The essential tool is the Hurwitz action of the braid group on the free group restricted to the braid monodromy associated to the truncated versal unfolding, that was first studied in depth by Catanese and Wajnryb [2] in the case of simple singularities of type *A*.

Relations (iv) on the contrary are due to degenerations along the hypersurface $x_0=0$, while those in (v) originate in the action of the group of equivalences.

The present paper should be viewed as a substantial contribution to the understanding of families of smooth elliptic surfaces and their monodromies.

Moduli spaces enter the stage, since they provide the appropriate means to study all families of a specified kind at once. In particular all their monodromy maps should assemble into a monodromy homomorphism defined on the orbifold fundamental group of the moduli space, so we taste a bit of ’stacky flavour’.

A particular nice example—which motivated our research—is provided by the families of elliptic curves, where the homological monodromies assemble into an isomorphism from the orbifold fundamental group of the quotient ${\mathbb {H}}/SL_2{{\mathbb {Z}}}$ to the automorphism group $SL_2{{\mathbb {Z}}}$ of the first homology of a curve, cf. Theorem 3. Our aim is to investigate possible generalisations to the case of families of elliptic surfaces, which we believe to be tractable and still to exhibit many characteristic features of the surface case in general.

A major difference from the curve case is the existence of—at least—three distinct moduli problems for families of elliptic surfaces which attract our attention:

(i) for smooth regular elliptic surfaces with a section. The coarse moduli space has been constructed by Miranda as the moduli space of Weierstrass fibrations with at most rational double points, cf. [10].
(ii) for smooth elliptic surfaces with a section and irreducible fibres only, equivalently for surfaces with a smooth Weierstrass model. That case is an instance of a moduli problem for polarised elliptic surfaces as considered by Seiler [12].
(iii) for smooth elliptic surfaces with a section and nodal fibres only, which were considered in [5] for the benefit of allowing a special kind of monodromy, cf. below.

To hope for as nice a result as in the elliptic curve case, we are forced to adjust the choice of monodromy to the choice of moduli problem. An educated guess among some natural monodromies leads to the following tentative list:

(i) algebraic or geometric monodromy. It takes values in the automorphism group of integral homology respectively the group of isotopy classes of diffeomorphism.
(ii) symplectic monodromy. Both the ambient space and the polarisation may be employed to construct a symplectic connection. The monodromy then takes values in the group of symplectic isotopy classes of symplectomorphisms.
(iii) bifurcation braid monodromy. We exploit the fact that families of elliptic surfaces with nodal fibres only naturally give rise to continuous families of finite sets in the base. Thus in case of regular surfaces the monodromy takes values in the braid group of the two-sphere, cf. [5].

Since symplectic monodromy remains quite mysterious despite the efforts of Seidel and others to enlighten the structure of symplectomorphism groups we have proposed a replacement of (ii) of a more topological flavour:

(ii’) braid class monodromy: Obtained from braid monodromy by imposing just as many relations on the image of braid monodromy as to make sure that it is well defined on the larger moduli space.

In any case it is desirable to understand the topological fundamental groups of the moduli quotients and the target groups of the monodromies. While our previous contributions were to monodromies in case (i) and (iii), the present paper yields the fundamental group in case (ii).

Our results also prepare the ground to handle the fundamental group in the other cases. To address (iii) we have to discard some parts of the moduli quotient. On the level of discriminant complements this corresponds to taking the bifurcation divisor into account, the set of parameters *u*, such that the projection of the corresponding hypersurface ${\mathcal {H}}_u$ to ${\mathbf {P}}^1$ is non-generic.

For case (i) on the other hand, we need to glue in some orbifold divisor to account for some families which are allowed in addition. The associated coarse space is naturally the coarse moduli space of elliptic surfaces with a section constructed as a moduli space of Weierstrass fibrations with at most rational double point singularities. To construct the appropriate stack structure over that space, to get the actual moduli stack for families of smooth elliptic surfaces, is an open challenge.

Of course we can initiate an analogous program in higher dimension. For example our new result may be extended along the lines of [6]. Nevertheless we should note a number of potential obstacles:

(i) In higher dimension a generalised bifurcation monodromy can be assigned as long as we admit only family of Weierstrass fibrations with generic bifurcation set of their fibrations. However this monodromy maps only to a group detecting the braiding in ${\mathbf {P}}^n$ of the critical loci, which then are positive dimensional and singular, cf. the interpretation of $\pi _1({\mathcal {U}}_{{\mathbf {P}}^n\!\!,d})$ as group of braiding in ${\mathbf {P}}^n$, [6].
(ii) Admitting also families of smooth Weierstrass fibrations, the need of a bifurcation class monodromy has to be checked and—if necessary—relations have to be imposed on the image of bifurcation monodromy.
(iii) A suitable relation of smooth elliptic fibrations with section to Weierstrass fibrations with mild singularities is needed for any progress on geometric monodromy.

## Zariski arguments

The ideas of Zariski provide the tool to get hold of a presentation for the fundamental group of divisor complements. While the relations need considerably more care, generators come with a distinct geometric flavour.

### Definition 2.1

Any element in a fundamental group of the complement of a divisor which can be represented by a path isotopic to the boundary of a small disc transversal to the divisor is called a *geometric element*.

In case of a punctured disc or affine line a free basis for the fundamental group is called a *geometric basis* if it consists of an ordered sequence of geometric elements simultaneously represented by paths only meeting in the base point, such that the product in descending order is homotopic to the boundary of the disc.

The discriminant complement ${\mathcal {U}}_{d}$ lies in the affine chart of ${\mathbf {P}}V_{n,d}$ given by $u^{\circ }{\ne 0}$. Thus ${\mathcal {U}}_{d}$ can be considered as a complement in the affine space ${\mathbb {C}}^N$, $N=6d+3$ with coordinates $u_\nu , u'_\nu , u''_\nu$ to the discriminant divisor ${\mathcal {D}}_{d}$.

We distinguish by an additional new notation $z:=u_{{3d}}$ and consider projections, along the coordinate *z* and along the coordinates with $\nu >0$ respectively:

$$\begin{aligned} p_z: {\mathbb {C}}^{N} \quad \longrightarrow \quad {\mathbb {C}}^{N-1},\qquad p_0: {\mathbb {C}}^{N} \quad \longrightarrow \quad {\mathbb {C}}^{3} \end{aligned}$$

together with the induced projection

$$\begin{aligned} {\bar{p}}_0: {\mathbb {C}}^{N-1} \quad \longrightarrow \quad {\mathbb {C}}^3 \end{aligned}$$

We denote by ${\bar{{\mathcal {A}}}}$ the discriminant locus in the parameter space ${\mathbb {C}}^3$ for the polynomial

$$\begin{aligned} y^3+u''_0y^2+u'_0 y+ u_0 \end{aligned}$$

and by ${\mathcal {B}}$ the bifurcation locus in the truncated parameter space ${\mathbb {C}}^{N-1}$. ${\mathcal {B}}$ is defined by the discriminant polynomial of a polynomial $q_d$ with respect to the variable *z* if $q_d$ defines the discriminant ${\mathcal {D}}_{d}$ in ${\mathbb {C}}^N\!$. Their pre-images are denoted by

$$\begin{aligned} {\mathcal {A}}={\bar{p}}_0^{-1}({\bar{{\mathcal {A}}}}),\quad {\hat{{\mathcal {A}}}}=p_z^{-1}({\mathcal {A}})=p_0^{-1}({\bar{{\mathcal {A}}}}), \quad \text { and }\quad {\hat{{\mathcal {B}}}}=p_z^{-1}({\mathcal {B}}) \end{aligned}$$

Note that points on ${\mathcal {B}}$ correspond to polynomials with degenerate or multiple critical values, while points on ${\mathcal {A}}$ correspond to polynomials with a critical value at infinity. In particular, ${\mathcal {A}}$ is the locus where the leading coefficient of $q_d$ considered as a polynomial in *z* vanishes.

### Lemma 2.2

Suppose *L* is a fibre of the projection $p_z$ such that its intersection ${\mathcal {D}}_L$ with the discriminant ${\mathcal {D}}$ consists of $\deg _z q_d$ points, then there is a split exact sequence

$$\begin{aligned} 1\rightarrow \pi _1(L-{\mathcal {D}}_L)\rightarrow \pi _1({\mathbb {C}}^N-{\hat{{\mathcal {A}}}}-{\hat{{\mathcal {B}}}}-{\mathcal {D}}) \rightarrow \pi _1({\mathbb {C}}^{N-1}-{\mathcal {A}}-{\mathcal {B}})\rightarrow 1 \end{aligned}$$

with a splitting map which takes geometric elements associated to ${\mathcal {B}}$ to geometric elements associated to ${\hat{{\mathcal {B}}}}$.

### Proof

In fact over the complement of ${\mathcal {A}}\cup {\mathcal {B}}$ the discriminant is a finite topological cover and its complement is a locally trivial fibre bundle with fibre the affine line punctured at $\deg _zp_{n,d}$ points. The exact sequence is now obtained from the long exact sequence of that fibre bundle. Exactness on the left follows from the fact that no free group of rank more than one admits a normal abelian subgroup.

To find a splitting map, we pick for each set of parameters in ${\mathbb {C}}^{N-1}$ a real upper bound for the moduli of all zeroes of the corresponding polynomial. This bound can be chosen continuously outside the zero set of the leading coefficient and thus defines a topological section and its induced splitting map over the complement of ${\mathcal {A}}$.

The final observation is that this topological section maps boundaries of small discs transversal to ${\mathcal {B}}$ to boundaries of small discs transversal to ${\hat{{\mathcal {B}}}}$ and disjoint to any other divisor. $\square$

### Remark 1

We should nevertheless note that boundaries of arbitrarily small discs transversal to ${\mathcal {A}}$ are mapped to boundaries of discs transversal to ${\hat{{\mathcal {A}}}}$ but also intersecting ${\mathcal {D}}$.

The group in the middle is hence determined as the semi-direct product of the other two by a map of $\pi _1({\mathbb {C}}^{N-1}-{\mathcal {A}}-{\mathcal {B}})$ to the automorphism group of $\pi _1(L-{\mathcal {D}}_L)$.

This has an immediate corollary on the level of presentations:

### Lemma 2.3

Suppose there is a presentation for the fundamental group of the base

$$\begin{aligned} \pi _1({\mathbb {C}}^{N-1}-{\mathcal {A}}-{\mathcal {B}})\quad \cong \quad \langle r_\alpha \mid {\mathcal {R}}_q\rangle \end{aligned}$$

with geometric generators $r_\alpha$, $\alpha$ from a suitable index set. Then there is a presentation

$$\begin{aligned} \pi _1({\mathbb {C}}^N-{\hat{{\mathcal {A}}}}-{\hat{{\mathcal {B}}}}-{\mathcal {D}}) \quad \cong \quad \langle t_i,{\hat{r}}_\alpha \mid {\hat{r}}_\alpha t_i^{^{-1}}{\hat{r}}_\alpha ^{^{-1}}\phi _\alpha (t_i),{\mathcal {R}}_q\rangle . \end{aligned}$$

where $\phi _\alpha$ denotes the automorphism associated to $r_\alpha$, the $t_i$ form a free geometric basis for a generic vertical line *L* punctured at $L\cap {\mathcal {D}}$, and the ${\hat{r}}_a$ are lifts of the $r_a$ by the topological section.

### Lemma 2.4

Suppose there is a presentation for the fundamental group of ${\mathbb {C}}^{N-1}-{\mathcal {A}}$

$$\begin{aligned} \pi _1({\mathbb {C}}^{N-1}-{\mathcal {A}})\quad \cong \quad \langle r_a \mid {\mathcal {R}}_a\rangle \end{aligned}$$

in terms of geometric generators $r_a$ in the complement of ${\mathcal {B}}$ and that $\pi _1(F - {\mathcal {B}}_F)$ is generated by geometric elements $r_b$. Then there is a presentation

$$\begin{aligned} \pi _1({\mathbb {C}}^N-{\hat{{\mathcal {A}}}}-{\mathcal {D}}) \quad \cong \quad \langle t_i,{\hat{r}}_a \mid t_i^{^{-1}}\phi _b(t_i),{\hat{r}}_a t_i^{^{-1}}{\hat{r}}_a^{^{-1}}\phi _a(t_i),{\mathcal {R}}_a\rangle , \end{aligned}$$

where $\phi _a$ (resp. $\phi _b$) is the automorphism associated to $r_a$ (resp. $r_b$), $t_i$ is a free geometric basis of $\pi _1(L-{\mathcal {D}}_L)$, and the ${\hat{r}}_a$ are lifts of $r_a$ by the topological section.

### Proof

The given information can be used with Lemma 1.5C of Nori [11] that associates an exact sequence with the projection ${\bar{p}}_0$

$$\begin{aligned} \pi _1(F - {\mathcal {B}}_F) \quad \rightarrow \quad \pi _1({\mathbb {C}}^{N-1} -{\mathcal {A}}- {\mathcal {B}}) \quad \rightarrow \quad \pi _1({\mathbb {C}}^3-{\bar{{\mathcal {A}}}})\quad \rightarrow \quad 1 \end{aligned}$$

if the image of the fibre *F* is generic in the base.

If we denote by ${\mathcal {N}}$ the subgroup generated by the $r_b$, i.e. the image of $\pi _1(F-{\mathcal {B}}_F)$, then a presentation can be given in the following form

$$\begin{aligned} \pi _1({\mathbb {C}}^{N-1} -{\mathcal {A}}- {\mathcal {B}}) \quad = \quad \langle r_a,r_b \mid {\mathcal {R}}_b, r_ar_b r_a^{^{-1}}\in {\mathcal {N}}, {\mathcal {R}}_a\subset {\mathcal {N}}\rangle \end{aligned}$$

Here an element is denoted to be in ${\mathcal {N}}$ if this element is a relation up to multiplication by a suitable element of ${\mathcal {N}}$ on the right.

In the next step we use Lemma 2.3 to get a corresponding presentation with ${\hat{{\mathcal {N}}}}$ the subgroup generated by the lifts ${\hat{r}}_b$.

$$\begin{aligned} \pi _1({\mathbb {C}}^N-{\hat{{\mathcal {A}}}}-{\hat{{\mathcal {B}}}}-{\mathcal {D}}) \quad \cong \quad \langle t_i,{\hat{r}}_\alpha ,{\hat{r}}_\beta \mid {\mathcal {R}}_b, {\hat{r}}_a{\hat{r}}_b {\hat{r}}_a^{^{-1}}\in {\hat{{\mathcal {N}}}}, {\mathcal {R}}_a\subset {\hat{{\mathcal {N}}}},\, {\hat{r}}_\alpha t_i^{^{-1}}{\hat{r}}_\alpha ^{^{-1}}\phi _\alpha (t_i) \rangle \end{aligned}$$

To get the presentation of $\pi _1({\mathbb {C}}^N-{\hat{{\mathcal {A}}}}-{\mathcal {D}})$, it suffices to drop the ${\hat{r}}_b$ from generators and relations. Then ${\mathcal {R}}_b$ all become trivial. The same holds for the relations of the form $r_ar_b r_a^{^{-1}}\in {\mathcal {N}}$. Since ${\mathcal {N}}$ becomes trivial ${\mathcal {R}}_a$ enters the list of relations. The last kind simplifies if the $r_\alpha$ is a geometric element associated to ${\mathcal {B}}$ otherwise nothing is changed. $\square$

Restricting ${\mathbb {C}}^{N-1}-{\mathcal {A}}$ to a fibre *F* the same argument applies and establishes

$$\begin{aligned} \pi _1({\hat{F}} - {\mathcal {D}}_{{\hat{F}}}) \quad \cong \quad \langle t_i \mid t_i^{^{-1}}\phi _b(t_i)\, \rangle . \end{aligned}$$

The second kind of relations in the lemma come from the automorphisms $\phi _a$ associated to geometric generators $r_a$. Any such can be studied restricting the projection $p_z$ to the preimage ${\hat{T}}_a$ of a disc $T_a$ transversal to ${\mathcal {A}}$ such that $r_a$ is given as a path to the boundary of $T_a$, the boundary in positive direction and the initial path back.

Then there is an induced map

$$\begin{aligned} j_a: \pi _1(L- {\mathcal {D}}_L) \quad \longrightarrow \quad \pi _1( {\hat{T}}_a - {\mathcal {D}}_{{\hat{T}}_a}) \end{aligned}$$

which allows to give a preliminary description of the presentation we look for:

### Proposition 2.5

The fundamental group of ${\mathbb {C}}^N-{\mathcal {D}}$ has a presentation

$$\begin{aligned} \pi _1\quad \cong \quad \langle t_i \mid {\mathcal {R}}_F, {\mathcal {K}}_a\rangle . \end{aligned}$$

where

(i) the $t_i$ form a geometric basis of $\pi _1(L-{\mathcal {D}}_L)$,
(ii) $\langle t_i \mid {\mathcal {R}}_F \rangle \cong \pi _1({\hat{F}} - {\mathcal {D}}_{{\hat{F}}})$ induced by the identity on the geometric basis,
(iii) ${\mathcal {K}}_a$ is a union over all $r_a$ of sets normally generating $\ker j_a$.

### Proof

We deduce this lemma using the presentation of Lemma 2.4. By (i) and (ii) the first kind of relations can be replaced by ${\mathcal {R}}_F$.

Since each ${\hat{r}}_a$ is transversal to ${\hat{{\mathcal {A}}}}$ it must be equal to a geometric element ${\hat{r}}_a'$ for ${\hat{{\mathcal {A}}}}$ up to some factor expressible in terms of geometric elements for ${\mathcal {D}}$. In $\pi _1({\mathbb {C}}^N-{\mathcal {D}})$ the element ${\hat{r}}_a'$ is trivial and the element ${\hat{r}}_\alpha t_i^{^{-1}}{\hat{r}}_\alpha ^{^{-1}}\phi _\alpha (t_i)$ must be transformed accordingly, but for the claim it is sufficient that both of them belong to $\ker j_a$.

Finally we have to argue why the relations of the last kind are redundant. In fact, each of them corresponds to a closed path which is homotopically trivial in the complement of ${\mathcal {A}}$. Lifting by the topological section yields homotopically trivial paths in the complement of ${\hat{{\mathcal {A}}}}$. But then they correspond to relations in $\pi _1({\hat{F}} - {\mathcal {D}}_{{\hat{F}}})$ which are already accounted for by ${\mathcal {R}}_F$. $\square$

The claim of the proposition is of course only an intermediate step on our way to give a presentation of the fundamental group. Obviously we have to make the relations explicit in the sense that every relation is given in terms of the chosen generators only.

### Remark 2.6

We were very lax about the base points. They should be chosen in such a way that all maps of topological spaces are in fact maps of pointed spaces. (In particular in the presence of a topological section there is no choice left; in the fibre and in the total space the base point is the intersection of the section with the fibre and its projection to the base yields the base point there.)

## Brieskorn Pham unfolding

In this section one aim is to choose a distinguished set of generators for $\pi _1({\mathbb {C}}^N-{\mathcal {D}})$. We pick some distinguished fibres $L_v$ of the projection $p_z:{\mathbb {C}}^N\rightarrow {\mathbb {C}}^{N-1}$ along the variable *z* where in each case $L_v-{\mathcal {D}}_L$ can be equipped with a distinguished geometric basis by the method of Hefez and Lazzeri [4]. For later use in Sect. 4 we establish a relation between different such bases.

In the next step we observe that each fibre of ${\bar{p}}_0$ is the base of an unfolding of a weighted homogeneous isolated plane curve singularity topologically equivalent to a Brieskorn-Pham polynomial of multiplicity 3. We exploit our knowledge of its discriminant knot group—the fundamental group of the discriminant complement in any universal unfolding, to get a presentation of the corresponding group in a generic fibre of $p_0$.

### Hefez Lazzeri path system

First we want to describe a natural geometric basis for some fibres of the projection $p_z:{\mathbb {C}}^N\rightarrow {\mathbb {C}}^{N-1}$. Since we follow Hefez and Lazzeri [4] we will call such bases accordingly. We note first that fibres $L_u$ of the projection correspond to affine pencils of polynomials

$$\begin{aligned} f_u(y,x)-z \end{aligned}$$

and their discriminant points ${\mathcal {D}}_L$ are exactly the *z* such that the *z*-level of $f_u$ is singular. As in [4] we restrict our attention to the linearly perturbed Brieskorn-Pham polynomial:

$$\begin{aligned} f= y^3-3v_0y+ x^{3d}- {3d}v_1 x. \end{aligned}$$

In that family the discriminant points for any generic pencil are in bijection to the elements in the bi-index set of cardinality $2({3d}-1)$:

$$\begin{aligned} I_{3d-1} =\{\,(i,j) \mid 1\le i<3,\,1\le j < {3d}\,\}. \end{aligned}$$

More precisely we get an expression for the critical values from [4]:

#### Lemma 3.1

(Hefez Lazzeri) The polynomial defining the critical value divisor is given by the expansion of the formal product ($\eta$ primitive root of order $3d-1$)

$$\begin{aligned} \prod _{(i,j) \in I_{3d-1}} \left( -z+2(-1)^{i}v_0^\frac{3}{2}+ ({3d}-1) \eta ^j v_1^{\frac{{3d}}{{3d}-1}}\right) . \end{aligned}$$

We deduce two immediate corollaries, that the discriminant sets are equal for suitably related parameter values and that they can be constructed inductively:

#### Lemma 3.2

The discriminant of the linearly perturbed polynomial *f* is invariant under the multiplication of $v_0$ by a third root of unity and of $v_1$ by a ${3d}$-th root of unity.

#### Proof

From the expansion above we see that the discriminant polynomial is a polynomial in $v_1^{\frac{{3d}}{{3d}-1}}$ but of course it is also a polynomial in $v_1$, hence it must be a polynomial in $v^{3d}_1$ since that is the least common power of both. Then it is obviously invariant under multiplying $v_1$ by a 3*d*-th root. The statement for $v_0$ is proved analogously. $\square$

#### Lemma 3.3

The critical values of *f* are distributed on circles of radius $({3d}-1)|v_1|^{\frac{{3d}}{{3d}-1}}$ centred around the critical values

$$\begin{aligned} \pm v_0^{\frac{3}{2}} \quad \text {of}\quad y^3-3v_0y. \end{aligned}$$

#### Proof

Again we can use lemma 3.1. A formal zero of the discriminant polynomial for *f* differs by a term $({3d}-1)v_1^{\frac{{3d}}{{3d}-1}}$ from a zero of the discriminant polynomial of $y^3-3v_0y$, and that difference is of the claimed modulus. $\square$

We assume now that $v_0,v_1$ are positive real and of sufficiently distinct magnitude

$$\begin{aligned} 0<v_1\ll v_0. \end{aligned}$$

**Fig. 2:**
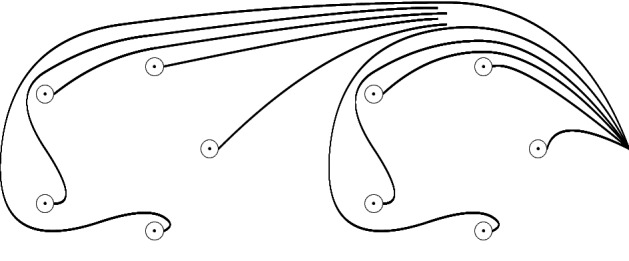
Hefez Lazzeri system in case ${3d}-1=5$

We define the Hefez Lazzeri geometric basis as indicated in Fig. 2 for ${3d}-1=5$, where each geometric generator is depicted as a tail and a loop around a critical value.

Of course the geometric element associated to a loop-tail pair is represented by a closed path based at the free end of the tail which proceeds along the tail, counterclockwise around the loop and back along the tail again.

For our convenience we introduce both a single and a double index notation for the geometric elements:

The $n={3d}-1$ elements of the base associated to punctures on the right are denoted by $t_{1,1},...,t_{1,n}$, respectively $t_1,\dots , t_{n}$ such that the corresponding critical values are enumerated counterclockwise starting on the positive real line. Similarly the remaining elements are denoted by $t_{2,1},...,t_{2,n}$, respectively $t_{n+1},\dots , t_{2n}$

The element $\delta _0$ represented by a path enclosing all critical values counter-clockwise deserves our special attention. We see immediately that as an element in the fundamental group it can be expressed as

3 $$\begin{aligned} \delta _0\quad = \quad t_{2n} t_{2n-1} \dots t_2 t_1 \end{aligned}$$

As we noticed in Lemma 3.2 the set of singular values remains unchanged upon multiplication the real $v_1$ by a ${3d}$-th root of unity $\xi$, resp. $v_0$ by a third root $\xi ^d$. The corresponding fibres are thus equipped with the same Hefez Lazzeri systems of paths.

We denote by $t_{i,j}(i',j')$, the elements of the Hefez-Lazzeri basis in the fibre at $v(i',j')=(v_0\xi ^{d(i'-1)}, v_1\xi ^{j'-1})$ and by $z_0$ the fibre coordinate of the Hefez Lazzeri base point, which may be assumed to belong to a topological section as in the proof of Lemma 2.2. We assume furthermore that this section is constant in all fibres we are looking at now.

Then we can compare the fundamental groups $\pi _1(L_{v(i',j')}-{\mathcal {D}}_{L_{i',j'}},(v(i',j'),z_0))$ along paths

$$\begin{aligned} \omega _{i',j'}:\quad s\,\mapsto \,(v_0\xi ^{sd(i'-1)},v_1\xi ^{s(j'-1)},z_0). \end{aligned}$$

#### Lemma 3.4

Conjugation by a path $\omega _{i',j'}$ induces an isomorphism

$$\begin{aligned} \omega ^*_{i',j'}:\pi _1(L_{v(i',j')}-{\mathcal {D}}_{L_{i',j'}},(v(i',j'),z_0))\rightarrow \pi _1(L_v-{\mathcal {D}}_{L_v},(v,z_0)) \end{aligned}$$

such that

$$\begin{aligned} \omega ^*_{i',j'}(t_{i',j'}(i',j'))= t_{1,1} \end{aligned}$$

#### Proof

We consider the case $i'=1$ first. Then along $\omega _{i',j'}$ all punctures move counterclockwise in the small discs covering an angle of $(j'-1)\vartheta$, $\vartheta =\frac{2\pi }{{3d}-1}$.

Accordingly each tail has to be adjusted alongside in slightly larger discs, but all other parts may just be kept fixed. In particular the tail segment with label $j'$ is moved to the segment with label 1.

For $i'=2, j'=1$ instead, the segment outside the small discs of each tail is affected as well. In this case the segments inside the two discs are moved to the corresponding segments in the other disc having the same label.

Combining the two moves we may conclude that the tail labeled by ${i',j'}$ is moved to the tail labeled by 1, 1 and so our claim holds. $\square$

For later use we should point out that the same consideration may be done for $v_0,v_1$ positive real, of sufficiently distinct magnitude, but

$$\begin{aligned} 0<v_0\ll v_1. \end{aligned}$$

which is like switching the roles of the variables *x* and *y*.

Now it is more convincing to describe the distribution of critical values as pairs on circles of radius centred at the ${3d}-1$ critical values of $x^{3d}-{3d}v_1 x$.

The elements in the corresponding geometric Hefez Lazzeri basis shall be denoted in the two-index notation as

$$\begin{aligned} t'_{1,1},t'_{1,2},\; t'_{2,1},t'_{2,2},\; \dots \;, t'_{{3d}-1,1},t'_{{3d}-1,2} \end{aligned}$$

see Fig. 3.

We also define the corresponding paths $\omega '_{j,j'}$ in this situation.

**Fig. 3:**
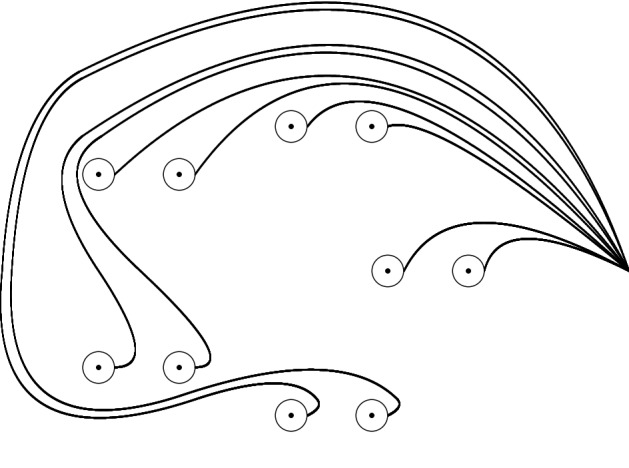
’dual’ Hefez Lazzeri system in case ${3d}-1=5$

### Brieskorn-Pham monodromy

The next aim is to determine the set of relations imposed on Hefez-Lazzeri generators by the geometric generators associated to ${\mathcal {B}}$. By Sect. 2 we get hold of this information if only we can determine a presentation for the discriminant complement in a generic fibre ${\hat{F}}$ of $p_0$.

To arrive there, we will compare the generic fibre *F* of ${\bar{p}}_0$ with the—possibly non-generic—fibre $F_{\!B\!P}$ which contains the Brieskorn-Pham polynomial

$$\begin{aligned} f_{\!B\!P}= y^3 + x^{3d} \end{aligned}$$

and both of them with the universal unfoldings of $f_{\!B\!P}$, respectively the weighted homogeneous polynomial $f_{gen}$ in the fibre *F*, which are both affine germs ${\mathbb {C}}^\mu$ of dimension $\mu =6d-2$.

#### Proposition 3.5

Suppose ${\hat{F}}$ is a generic fibre of $p_0$ sufficiently close to ${\hat{F}}_{\!B\!P}$, then there is an isomorphism of fundamental groups

$$\begin{aligned} \pi _1({\mathbb {C}}^\mu -{\mathcal {D}}_{f_{\!B\!P}}) \quad \longrightarrow \quad \pi _1({\hat{F}} - {\mathcal {D}}_{{\hat{F}}}) \end{aligned}$$

induced by an identification of geometric bases.

#### Proof

We have to compose several maps on fundamental groups. First we compare the universal unfolding of the Brieskorn-Pham polynomial with its affine unfolding by the Brieskorn-Pham fibre ${\hat{F}}_{\!B\!P}$. The later is weighted homogenous with positive weights and therefore embeds its discriminant complement into the discriminant complement of the former. The corresponding map induced by the identification of a Hefez-Lazzeri geometric basis

$$\begin{aligned} \pi _1({\hat{F}}_{\!B\!P}- {\mathcal {D}}_{{\hat{F}}_{\!B\!P}}) \quad \longrightarrow \quad \pi _1({\mathbb {C}}^\mu -{\mathcal {D}}_{f_{\!B\!P}}) \end{aligned}$$

is an isomorphism, since in Section 4.3 of [6] it was shown, that for the complement ${\hat{F}}_{\!B\!P}- {\mathcal {D}}_{{\hat{F}}_{\!B\!P}}$ the general plane section has only cusps and nodes and is isotopic to that of the complement ${\mathbb {C}}^\mu -{\mathcal {D}}_{f_{\!B\!P}}$, cf. the proof of Prop.4.10 *loc.cit*.

Similarly, we get an induced map from the fundamental group of the discriminant complement of the affine unfolding of a non-Brieskorn-Pham weighted homogeneous polynomial $f_{gen}$ to that of its universal unfolding:

$$\begin{aligned} \pi _1({\hat{F}}- {\mathcal {D}}_{{\hat{F}}}) \quad \longrightarrow \quad \pi _1({\mathbb {C}}^\mu -{\mathcal {D}}_{f_{gen}}) \end{aligned}$$

This map is obviously surjective, since both groups are generated by a geometric basis in any shared generic fibre over *F*, since the latter can again be considered an subspace of the truncated universal unfolding.

Since ${\hat{F}}_{\!B\!P}$ and ${\hat{F}}$ are fibres of the projection ${\bar{p}}_0$, the latter generic and sufficiently close, we get an induced map

$$\begin{aligned} \pi _1({\hat{F}}_{\!B\!P}- {\mathcal {D}}_{{\hat{F}}_{\!B\!P}}) \quad \longrightarrow \quad \pi _1({\hat{F}}- {\mathcal {D}}_{{\hat{F}}}) \end{aligned}$$

which again is surjective, since it commutes with a surjective map of a geometric basis onto both sides.

Composing these maps in a suitable way, we get a surjective map induced by an identification of geometric bases:

$$\begin{aligned} \pi _1({\mathbb {C}}^\mu -{\mathcal {D}}_{f_{\!B\!P}}) \quad \rightarrow \quad \pi _1({\hat{F}}_{\!B\!P}- {\mathcal {D}}_{{\hat{F}}_{\!B\!P}}) \quad \rightarrow \quad \pi _1({\hat{F}}- {\mathcal {D}}_{{\hat{F}}}) \quad \rightarrow \quad \pi _1({\mathbb {C}}^\mu -{\mathcal {D}}_{f_{gen}}) \end{aligned}$$

But the composition is known by [8] to be an isomorphism, hence all maps are, in particular the composition we need for the claim. $\square$

The explicit relations for the discriminant knot group has been proved elsewhere and are cited here to be used in the proof of the main theorem in Sect. 4.2.

#### Theorem 5

[7] The fundamental group of the discriminant complement in a versal unfolding of a Brieskorn-Pham polynomial $x^3+y^{\ell +1}$ is presented by

$$\begin{aligned} \left\langle t_{\varvec{i}},\, {\varvec{i}}\in I_\ell \quad \Bigg | \begin{array}{rccl} &{} &{} t_{\varvec{i}}t_{\varvec{j}}=t_{\varvec{j}}t_{\varvec{i}}, &{}({\varvec{i}},{\varvec{j}})\not \in E,\\ &{}&{} t_{\varvec{i}}t_{\varvec{j}}t_{\varvec{i}}=t_{\varvec{j}}t_{\varvec{i}}t_{\varvec{j}}, &{} ({\varvec{i}},{\varvec{j}})\in E,\\ &{}&{} t_{\varvec{i}}t_{\varvec{k}}t_{\varvec{j}}t_{\varvec{i}}=t_{\varvec{j}}t_{\varvec{i}}t_{\varvec{k}}t_{\varvec{j}}, &{} ({\varvec{i}},{\varvec{j}}),({\varvec{i}},{\varvec{k}}),({\varvec{j}},{\varvec{k}})\in E \end{array} \right\rangle \end{aligned}$$

Here *E* consists of the oriented edges $({\varvec{i}},{\varvec{j}})$ with ${\varvec{i}}<{\varvec{j}}$ of $\Gamma _\ell$.

Both the lexicographical order on double indices and the total order on the single indices define the same set of oriented edges.

#### Remark 2

While the first two kinds of relations are insensitive to the order of the indices, the last row is not, so the appropriate condition , $$i< j < k $$ on the indices, has to be added if we want to make our statement in terms of the edges of the unoriented graph $$\Gamma _\ell $$.

## Asymptotes

In this section we get the explicit relations imposed by the degenerations along the divisor ${\mathcal {A}}$. The local model, that is the restriction of the projection along *z* to the preimage of a small disc transversal to ${\mathcal {A}}$ is well understood. In fact a presentation for its fundamental group can be given in terms of a geometric basis in a local reference fibre subjected to a single relation.

The problem—which lies at the heart of our argument—is to bring those local relations together globally: Basically we can start with a path connecting a global reference fibre to a local disc transversal to ${\mathcal {A}}$. The trivialisation along the path induces an isomorphism between the fundamental groups of the local and global reference fibres at the endpoints.

In practice we are just able to determine unambiguously the images of local relations along a very restricted set of paths, which we have to construct with great care.

But we succeed to do so, that a geometric element associated to ${\mathcal {A}}$ comes naturally with each path and the boundary of the corresponding local disc. In fact it is our final task for this section to show that the geometric elements thus obtained suffice to apply the results of Sect. 3.

### Critical points of a distinguished subfamily

In this subsection we consider a suitable family ${\mathcal {G}}$ of polynomials *f* in ${\mathbb {C}}[x,y]$ depending on four complex parameters $\uplambda _0,\uplambda _1,\varepsilon ,\uplambda$.

$$\begin{aligned} \begin{array}{cccl} f &{} = &{} &{} y^3-3\uplambda _0y + x^{3d}-{3d}\uplambda _1x \\ &{}&{} - &{} \frac{3d}{3d-1}\varepsilon x^{3d-1} - 3\uplambda \uplambda _0y x^{2d} \end{array} \end{aligned}$$

Our aim is to get information on how the critical values depend on the parameters. So we first simplify the expression for *f* on the set of critical points, ie. the set of points with vanishing gradient.

#### Lemma 4.1

The value of *f* at critical points is given by

4 $$\begin{aligned} f|_{\nabla f=0} = -2\uplambda _0y - (3d-1) \uplambda _1x - {\textstyle \frac{1}{3d-1}}\varepsilon x^{3d-1} \end{aligned}$$

#### Proof

First the vanishing gradient condition may be expressed by the following pair of equations:

5 $$\begin{aligned} y^2= & {} \uplambda _0(1+\uplambda x^{2d})\nonumber \\ x^{3d-1}= & {} \uplambda _1+ \varepsilon x^{3d-2} + 2\uplambda \uplambda _0y x^{2d-1} \end{aligned}$$

Next we use them to replace the pure monomials in *f*:

$$\begin{aligned} f= & {} y \uplambda _0(1+\uplambda x^{2d}) -3\uplambda _0y \\&+x(\uplambda _1+ \varepsilon x^{3d-2}+ 2\uplambda \uplambda _0y x^{2d-1} )\\&-{3d}\uplambda _1x-{\textstyle \frac{3d}{3d-1}}\varepsilon x^{3d-1} -3\uplambda \uplambda _0y x^{2d} \\= & {} -2\uplambda _0y -(3d-1) \uplambda _1x -{\textstyle \frac{1}{3d-1}}\varepsilon x^{3d-1} .\\ \end{aligned}$$

$$\square $$

#### Lemma 4.2

Suppose $\uplambda _0^3\in {\mathbb {R}}^+$, $\uplambda _1^{3d}\in {\mathbb {R}}^+$ and $\varepsilon \in {\mathbb {R}}^+$. Then there is a positive real $\uplambda _{\text {crit}}$ such that the number of critical points (counted with multiplicity) is maximal for $\uplambda \in [0,\uplambda _{\text {crit}}[$ and drops by one at $\uplambda =\uplambda _{\text {crit}}$.

#### Proof

From the Eq. (5) we deduce a polynomial equation for the *x*-coordinate of all critical points. To eliminate *y* we note, that the following expression

$$\begin{aligned} \big (x^{3d-1}-\uplambda _1-\varepsilon x^{3d-2}- 2\uplambda \, \uplambda _0y x^{2d-1} \big ) \big (x^{3d-1}-\uplambda _1-\varepsilon x^{3d-2} + 2\uplambda \, \uplambda _0y x^{2d-1} \big ) \end{aligned}$$

is zero on critical points by the second equation of (5). Due to the invariance under $y\mapsto -y$, our expression is a polynomial in $y^2$ and *x*. We may thus insert the right hand side of the first equation of (5) to get a polynomial in *x* only.

$$\begin{aligned} \big (x^{3d-1}-\uplambda _1-\varepsilon x^{3d-2}\big )^2- \big (2\uplambda \, \uplambda _0x^{2d-1} \big )^2\uplambda _0(1+\uplambda x^{2d}) \end{aligned}$$

We easily extract the leading and subleading coefficient

$$\begin{aligned} 1-4\uplambda ^3\uplambda _0^3,\quad -2 \varepsilon \end{aligned}$$

Let $\uplambda _{\text {crit}}$ be the positive real root in $\uplambda$ of the leading coefficient. Then on the positive real axis the algebraic number of critical points drops only at $\uplambda _{\text {crit}}$. Moreover the next coefficient is non-zero at $\uplambda =\uplambda _{\text {crit}}$, therefore it drops by one only. $\square$

#### Lemma 4.3

Suppose $\varepsilon , \uplambda _1\in {\mathbb {R}}^{\ge 0}$, $\uplambda _0\ne 0$. Then there is no degenerate critical point with $\uplambda _0y$, $x \in {\mathbb {R}}^+$, $\uplambda \in [0,\uplambda _{\text {crit}}[$, except if all, $\uplambda ,\uplambda _1$ and $\varepsilon$, vanish.

#### Proof

It suffices to show that the gradient vectors to the equations (5) are linearly independent at solutions with $\uplambda _0y, x \in {\mathbb {R}}^+$.

$$\begin{aligned} \begin{vmatrix} 2y&-2d \uplambda \uplambda _0x^{2d-1}\\ -2\uplambda \uplambda _0x^{2d-1}&\begin{array}{c} (3d-1)x^{3d-2} -(3d-2)\varepsilon x^{3d-3}\\ -2(2d-1) \uplambda \uplambda _0yx^{2d-2} \end{array} \end{vmatrix}\ne & {} 0\\ \end{aligned}$$

We multiply the first column by *y*, the second by *x*, both of which are non-vanishing by assumption, to get an equivalent claim:

$$\begin{aligned} \begin{vmatrix} 2y^2&-2d \uplambda \uplambda _0x^{2d}\\ -2\uplambda \uplambda _0yx^{2d-1}&\begin{array}{c} (3d-1)x^{3d-1} -(3d-2)\varepsilon x^{3d-2}\\ -2(2d-1) \uplambda \uplambda _0yx^{2d-1} \end{array} \end{vmatrix}\ne & {} 0\\ \end{aligned}$$

Next we apply the Eq. (5) and simplify the entry in the right bottom corner

$$\begin{aligned} \begin{vmatrix} 2\uplambda _0(1+\uplambda x^{2d})&-2d \uplambda \uplambda _0x^{2d}\\ -2\uplambda \uplambda _0y x^{2d-1}&\begin{array}{c} (3d-1)\uplambda _1+\varepsilon x^{3d-2}\\ +2d \uplambda \uplambda _0yx^{2d-1} \end{array} \end{vmatrix}\ne & {} 0\\ \end{aligned}$$

We divide the first row by $\uplambda _0$ and add *d* times the first column to the last column:

$$\begin{aligned} \begin{vmatrix} 2(1+\uplambda x^{2d})&2d\\ -2\uplambda \uplambda _0y x^{2d-1}&(3d-1)\uplambda _1+\varepsilon x^{3d-2} \end{vmatrix}\ne & {} 0\\ \end{aligned}$$

From the hypothesis it is now easy to deduce that both summands of the determinant are non-negative real, and at least one summand is positive. $\square$

#### Proposition 4.4

Suppose $\varepsilon \in {\mathbb {R}}^{\ge 0},\uplambda _0^3,\uplambda _1\in {\mathbb {R}}^+$. Then for $\uplambda \in [0,\uplambda _{\text {crit}}[$ there is a unique critical point $({\check{x}},{\check{y}})$ with $\uplambda _0{\check{y}}, {\check{x}}\in {\mathbb {R}}^+$.

Moreover its critical value bounds the modulus of all other critical values, strictly for $\varepsilon >0$.

#### Proof

Let us rewrite the Eq. (5) in the following way:

$$\begin{aligned} \uplambda _0^2y^2= & {} \uplambda _0^3(1+\uplambda x^{2d}) \\ x^{3d-1}= & {} \uplambda _1+ \varepsilon x^{3d-2} + 2\uplambda \uplambda _0y x^{2d-1} \end{aligned}$$

For $\uplambda =0$ there is a unique solution with $\uplambda _0y,x \in {\mathbb {R}}^+$, because in that case the second equation has a unique solution ${\check{x}}$ in ${\mathbb {R}}^+$ by the sign rule.

Since solutions depend continuously on the parameter $\uplambda$, there are only the following transitions, which lead to a change of the number of positive real solutions:

(i) a solution tends to infinity, which actually happens for the critical parameter, but nowhere else on the interval $[0,\uplambda _{\text {crit}}]$ by Lemma 4.2
(ii) positive real solutions become semi-positive or vice versa, but with the given hypotheses there is never a semi-positive real solution, since $x=0$ implies $\uplambda _1=0$ and $\uplambda _0y=0$ implies $\uplambda _0=0$ or $x^{2d}<0$.
(iii) positive real solutions become complex and vice versa, but also this case can be excluded: Suppose a complex solution tends to a positive real solution, then there is another complex solution tending to the same real solution, since both equations are real and complex conjugation acts on solutions. Therefore the limit solution is positive real and degenerate. But with the given hypotheses such degenerate solutions do not exit due to Lemma 4.3.

The uniqueness claim is thus established. To prove the maximality claim for the value of the distinguished solution ${\check{x}}, {\check{y}}$, we deduce some further properties from our equations

$$\begin{aligned} \uplambda _0^2y^2= & {} \uplambda _0^3(1+\uplambda x^{2d}) \\ x^{3d-1}= & {} \uplambda _1+ \varepsilon x^{3d-2} + 2\uplambda \uplambda _0y x^{2d-1} \end{aligned}$$

(i) a critical point with *x* of smaller modulus than ${\check{x}}$ has - by the first equation - also $|\uplambda _0y|<\uplambda _0{\check{y}}$ and hence smaller critical value by Lemma 4.1.
(ii) for $\uplambda =0$ the distinguished critical point has maximal ${\check{x}}$ and uniquely so if $\varepsilon >0$.
(iii) For $\varepsilon \in {\mathbb {R}}^+$ suppose $\uplambda _0y,x$ is a critical point with $|x|={\check{x}}$. Then
  $$\begin{aligned} |x^{3d-1}|= {\check{x}}^{3d-1}= & {} \uplambda _1+ \varepsilon {\check{x}}^{3d-2} + 2 \uplambda \uplambda _0{\check{y}} {\check{x}}^{2d-1}\\= & {} \uplambda _1+ \varepsilon |x^{3d-2}| + 2 \uplambda \uplambda _0{\check{y}} |x^{2d-1}| \end{aligned}$$
  By the first equation above $|\uplambda _0y| \le \uplambda _0{\check{y}}$. Then comparing the second equation with the equation for the modulus we deduce that all summands in the second equation are positive real. In particular $x^{3d-1},x^{3d-2}\in {\mathbb {R}}^+$ and so $x={\check{x}}$ and thus $\uplambda _0y =\uplambda _0{\check{y}}$. Hence there is no second critical point $\uplambda _0y,x$ with $|x|={\check{x}}$.

By the continuity of critical points we may conclude, that for all admissible $\uplambda$ and $\varepsilon$ the last coordinate of a critical point is bounded by that of the distinguished critical point. Therefore the distinguished critical points has maximal value according to the first observation. $\square$

### Asymptotic arcs, paths, and induced paths

We consider arcs $\alpha _0,\alpha _1$ in the parameter space of the family ${\mathcal {G}}$ starting at the Hefez-Lazzeri base point $\uplambda _0=v_0,\uplambda _1=v_1,\,\varepsilon =0,\,\uplambda =0,$ which corresponds to a polynomial $f=y^3-3 v_0 y+ x_1^{3d}-{3d}v_1 x_1$ admitting a Hefez-Lazzeri geometric basis. They are composed of three parts each

(i) $\uplambda ,\varepsilon =0$, $\uplambda _1$ stays constant and $\uplambda _0$ moves from $v_0$ to $v_0 e^{j\frac{2\pi i}{3}}$ for the path $\alpha _j$, compare the construction of the paths $\omega$ in 3.1.
(ii) $\uplambda =0,\uplambda _0,\uplambda _1$ stay fixed, $\varepsilon$ increases to some small finite value.
(iii) $\uplambda _0,\uplambda _1,\varepsilon$ stay fixed, $\uplambda$ increases from 0 to $\uplambda _{\text {crit}}$.

By Lemma 4.2 each $\alpha _j$ leads to a point of ${\mathcal {A}}$ without intersecting ${\mathcal {A}}$ elsewhere, so we get well defined geometric elements associated to ${\mathcal {A}}$. An arbitrarily small isotopy yields the same geometric element represented by a path $\gamma _j$ in the complement of ${\mathcal {A}}\cup {\mathcal {B}}$.

#### Proposition 4.5

The relations imposed on the generators along the paths $\gamma _0, \gamma _1$ are

$$\begin{aligned} t_1(t_{1}^{^{-1}}\delta _0)^{{3d}-1}=(t_{1}^{^{-1}}\delta _0)^{{3d}-1}t_1, \quad t_{3d}(t_{{3d}}^{^{-1}}\delta _0)^{{3d}-1}=(t_{{3d}}^{^{-1}}\delta _0)^{{3d}-1}t_{3d}. \end{aligned}$$

#### Proof

By Lemma 4.2 the critical coordinate ${\check{x}}$ has a simple pole at $\uplambda _{\text {crit}}$. Hence by (5) the critical coordinate ${\check{y}}$ has a pole of order 2*d* and by (4) the corresponding critical value ${\check{z}}$ has a pole of order ${3d}-1$ at $\uplambda _{\text {crit}}$.

We consider a disc $\Delta$ which spans the noose of $\gamma$ and is transversal to ${\mathcal {A}}$. The discriminant complement restricted to this disc forms what may be called an *asymptotic model*, and is diffeomorphic to the complement in $\Delta \times {\mathbb {C}}$ of the zero set of

$$\begin{aligned} (z^{6d-3}-1) (\uplambda ^{{3d}-1}z-1),\quad |\uplambda |\ll 1 \end{aligned}$$

with $6d-2$ the vertical degree of the discriminant, the total number of critical points, and the second factor corresponding to the critical value ${\check{z}}$, the branch going to infinity with pole order ${3d}-1$ along ${\mathcal {A}}$.

In that situation [6, Prop.5.2] gives the relation $b^{{3d}-1}b'=b'b^{{3d}-1}$ if for some fibre there is $r>0$ such that

(i) a geometric generator associated to asymptotic branch at ${\check{z}}$ is supported outside the disc of radius *r*, which contains all other critical values of the fibre.
(ii) *b* is homotopic to the boundary of the disc of radius *r* and the product with $b'$ represents the element of a loop going around all critical values.

By Proposition 4.4 for $\uplambda$ real, $0<\uplambda _{\text {crit}}-\uplambda \ll 1$ there is $r>0$ with $0<{\check{z}}-r\ll 1$ such that *i*) holds. Moreover along the path $\alpha _0,\alpha _1$ the element $b'$ is identified with $t_1,t_{3d}$ resp., while the loop around all critical values is identified with $\delta _0$ along both arcs.

Hence the *b* to meet condition *ii*) is identified with $t_1^{^{-1}}\delta _0$ resp. $t_{3d}^{^{-1}}\delta _0$, and the claim of the proposition is proved. $\square$

To show that we need not consider more paths associated to ${\mathcal {A}}$ than $\gamma _0, \gamma _1$ we turn back to the projection

$$\begin{aligned} p_0: \; {\mathbb {C}}^{N-1} \quad \rightarrow \quad {\mathbb {C}}^3 \;=\; \{ (u''_0, u'_0, u_0) \} \end{aligned}$$

along all coefficients of monomials containing $x_0$ with positive exponent.

Then ${\mathcal {A}}$ is the pull back along $p_0$ of the discriminant locus ${\bar{{\mathcal {A}}}}$ for the polynomial

$$\begin{aligned} y^3+u''_0y^2+u'_0 y+ u_0 \end{aligned}$$

The parameter space of the family ${\mathcal {G}}$ projects to $\{(0, -3\uplambda \uplambda _0, 1) \mid \uplambda \in {\mathbb {C}}\}$, a line $L_{\mathcal {G}}$ of ${\mathbb {C}}^3$ transversal to ${\bar{{\mathcal {A}}}}$ and contained in the hyperplane $u''_0=0$.

#### Proposition 4.6

The paths $\gamma _0,\gamma _1$ project to generators of $\pi _1({\mathbb {C}}^3-{\bar{{\mathcal {A}}}})$.

#### Proof

Thanks to the Tschirnhaus transformation the complement of ${\bar{{\mathcal {A}}}}$ in ${\mathbb {C}}^3$ has a deformation retraction to the complement restricted to the hyperplane $u''_0=0$, which is the complement of a cuspidal cubic

$$\begin{aligned} {\mathbb {C}}^2 \,-\, C, \quad \text {with}\quad C \,=\,\{ (u'_0,u_0) \mid 27u_0^2-4 {u_0'}^3 \} \end{aligned}$$

It thus suffices to prove that the projected paths $\gamma '_0, \gamma '_1$ generate $\pi _1({\mathbb {C}}^{2}-C)$. In fact, together with a third path $\gamma '_2$ they form a geometric basis for the three intersection points of $L_{\mathcal {G}}$ with the cuspidal cubic *C* associated to the arcs

$$\begin{aligned} \alpha '_i = \{ (-3\uplambda \uplambda _0,1)\mid \uplambda \in [0,\uplambda _{\text {crit}}], \uplambda _0=\rho ^iv_0\} \end{aligned}$$

since the first two parts of the arcs $\alpha _0,\alpha _1$ project to (0, 0, 1) under $p_0$ and the last parts to $\alpha '_0,\alpha '_1$ respectively.

The braid group on three strands is isomorphic to $\pi _1({\mathbb {C}}^2-C)$ and tedious but elementary arguments identify the elements $\gamma '_0,\gamma '_1$ with the standard generators.

Hence generation also of $\pi _1({\mathbb {C}}^3-{\bar{{\mathcal {A}}}})$ follows. $\square$

Now it is possible to give a proof of

#### Theorem 6

The fundamental group $\pi _1({\mathcal {U}}_{d})$ is finitely presented with generators

$$\begin{aligned} t_1, \dots , t_{2n}, \quad n=3d-1 \end{aligned}$$

and relations of two kinds:

(1) Relations associated to the graph $\Gamma _n$:

$$\begin{aligned}&t_i t_j= t_j t_i&\text { if } (i,j)\not \in E_{d} \\&t_i t_j t_i= t_j t_i t_j&\text { if } (i,j)\in E_{d} \\&t_i t_j t_k t_i= t_j t_k t_i t_j&\text { if } \begin{array}[t]{ll} (i,j),(i,k),(j,k)\in E_{d} \\ i<j<k \end{array} \\ \end{aligned}$$

**Fig. 4:**
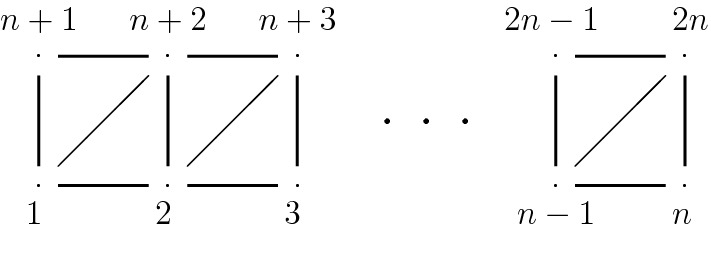
The graph $$\Gamma _n$$

(2) Relations associated to the asymptotic locus with $t_j=t_1$ and $t_j=t_{n+1}$ (Fig. 4):

$$\begin{aligned} \bigg ( t_j^{^{-1}}\Big ( t_{2n} t_{2n-1} \cdots t_2 t_1 \Big )\bigg )^{n} \,=\quad \bigg (\Big ( t_{2n} t_{2n-1} \cdots t_2 t_1 \Big ) t_j^{^{-1}}\bigg )^{n} \end{aligned}$$

#### Proof

The result is obtained collecting the ingredients for Prop. 2.5. First the generators $t_i$ are given by a geometric basis of Hefez-Lazzeri type in a fibre *L* of the map $p_z$.

The first set of relations may then be copied from Theorem 5, since it presents the fundamental group $\pi _1({\hat{F}}-{\mathcal {D}}_{{\hat{F}}})$ of a generic fibre of $p_0$ thanks to Proposition 3.5.

The second set of relations correspond to the geometric elements $\gamma _0,\gamma _1$ associated to ${\mathcal {A}}$. The elements of Proposition 4.5 normally generate the corresponding kernels, so it suffices to add these to elements. They were only slightly rewritten according to

$$\begin{aligned} t_j(t_{j}^{^{-1}}\delta _0)^{n}=(t_{j}^{^{-1}}\delta _0)^{n}t_j \quad \iff \quad \delta _0(t_{j}^{^{-1}}\delta _0)^{n-1}t_{j}^{^{-1}}=(t_{j}^{^{-1}}\delta _0)^{n} \end{aligned}$$

and the shorthand $\delta _0$ was replaced by its expression in the generators. $\square$

## Moduli quotient

Let us get now to the fundamental group of the moduli space ${\mathcal {M}}_{d}$ of smooth Weierstrass surfaces. Of course we have first to define the appropriate moduli problem and then to show how we can construct ${\mathcal {M}}_{d}$ as a quotient of the parameter space ${\mathcal {U}}_{d}'$ by a suitable group action.

### Definition 5.1

Suppose $W_1, W_2$ are Weierstrass surfaces. An isomorphism $\phi :W_1\rightarrow W_2$ is called an *isomorphism of Weierstrass surfaces*, if $\phi$ preserves the section at infinity and fits into a commutative diagram together with the elliptic fibrations

$$\begin{aligned} W_1&{\mathop {\longrightarrow }\limits ^{\phi }}&W_2\\ \downarrow&\downarrow \\ {\mathbf {P}}^1\longrightarrow & {} {\mathbf {P}}^1 \end{aligned}$$

Over the parameter space $$V_{d}'$$ there is the tautological Weierstrass surface $${\mathcal {W}}_{d}$$ in $$Y_d\times V_{d}'$$ defined by Eq. (2). The group $$G={\mathbb {C}}^*\times {\text {GL}}_{2}$$ acts on $$Y_d$$ and $$ V_{d}'$$ via

$$\begin{aligned} (\uplambda ,A)\cdot (\mathbf{x}, y_0,y,y_2) \quad = \quad (A^{^{-1}}\cdot \mathbf{x}, y_0, \uplambda ^2 y,\uplambda ^3 y_2). \\ (\uplambda ,A)\cdot (p,q)) \quad = \quad (\uplambda ^6 p{\circ } A, \uplambda ^4 q{\circ } A). \end{aligned}$$

where *p*, *q* are understood as elements in ${\text {Sym}}^{2d}({\mathbb {C}}^2)^\vee ={\mathbb {C}}[x_0,x_1]_{2d}$ and ${\mathbb {C}}[x_0,x_1]_{3d}$ resp. Note that both ${\mathcal {W}}_{d}$ and ${\mathcal {D}}_{n,d}'$ are invariant hypersurfaces for the corresponding action on their ambient spaces.

### Proposition 5.2

Every isomorphism of Weierstrass surfaces is induced from an automorphism in *G* of the tautological Weierstrass fibration ${\mathcal {W}}_{d}$.

### Proof

Given an isomorphism $\phi$ of Weierstrass surfaces we get immediately an induced automorphism of ${\mathbf {P}}^1$ and we may pick some $A\in {\text {GL}}_{2}$ inducing it.

Therefore we may assume without loss of generality, that $\phi$ induces the identity on the base. Hence $\phi$ induces abstract isomorphisms of plane cubic curves in Weierstrass normal form, mapping points at infinity to points at infinity.

Now it is well known, that each such isomorphism is induced by an automorphism of ${\mathbf {P}}^2$ of the form determined by a non-vanishing complex number $\uplambda$ acting on the coordinates by $\uplambda \cdot (y_0:y:y_2)=(y_0:\uplambda ^{2}y:\uplambda ^3 y_2)$.

Hence we get a map from ${\mathbf {P}}^1$ to a subgroup of ${\text {PGL}}_3$ isomorphic to ${\mathbb {C}}^*$. Thus it must be constant and our claim is proved. $\square$

According to this result it is natural to conceive the following definition:

### Definition 5.3

The Miranda moduli space ${\mathcal {M}}_{d}$ of smooth Weierstrass fibrations is the quotient of the base space ${\mathcal {U}}_{d}'$ by the induced action of *G*.

### Remark 5.4

The definition of the fundamental group has to take care of the fact, that moduli spaces do not parametrize isomorphism classes of objects, rather they represent isomorphism classes of families and hence pick up isotropy corresponding to automorphisms of objects.

### Proposition 5.5

There is an exact sequence

$$\begin{aligned} \pi _1({\mathbb {C}}^*\times {\text {GL}}_{2},1) \longrightarrow \pi _1({\mathcal {U}}_{d}, u),\longrightarrow \pi _1({\mathcal {M}}_{d},[u])\longrightarrow 1. \end{aligned}$$

where the action of ${\mathbb {C}}^*$ is defined by $\uplambda \cdot (p,q)=(\uplambda ^4p,\uplambda ^6q)$ and ${\text {GL}}_{2}$ acts by linear coordinate change $A\cdot (p,q)=(p{\circ } A,q{\circ } A)$.

### Proof

First we note that the affine group ${\mathbb {C}}[x_0,x_1]_d$ acts on $Y_d$ by

$$\begin{aligned} s(x_0,x_1)\cdot (x_0,x_1,y_0,y,y_2) \quad = \quad (x_0,x_1, y_0,y-s(x_0,x_1) y_0,y_2) \end{aligned}$$

The action is naturally extended to $Y_d\times V_{n,d}$ with ${\mathcal {W}}_{d}$ an invariant hypersurface such that the induced action on ${\mathcal {U}}_{d}$ is free and faithful and has ${\mathcal {U}}_{d}'$ as a transversal section. Hence we may replace $\pi _1({\mathcal {U}}_{d},u)$ by $\pi _1({\mathcal {U}}_{d}',u)$. But then the claim is obviously just the exact sequence for orbifold fundamental groups and the action of *G* on ${\mathcal {U}}_{d}$ inducing the action of *G* on ${\mathcal {U}}_{d}'$ made explicit. $\square$

In the following discussion we are going to combine results on fundamental groups of ${\mathcal {U}}_{d}$ and various of its subspaces which do *not* have the same base point.

Still all these base points are contained in a ball in ${\mathcal {U}}_{d}$, so our convention is that all occurring fundamental groups are identified using a connecting path for their base points inside this ball, which makes the identification unambiguous.

Let *u* now be the parameter point corresponding to the Brieskorn-Pham hypersurface

$$\begin{aligned} u:\quad y^3+x_0^{3d}+x_1^{3d}. \end{aligned}$$

Its ${\mathbb {C}}^*$-orbit belong entirely to the affine Brieskorn-Pham family ${\mathcal {F}}$ to which we shift our attention for the moment

$$\begin{aligned} y^3+a_0x_0^{3d}+a_1x_1^{3d}. \end{aligned}$$

### Definition 5.6

The elements $\delta _0,\delta _1$ are defined as the elements represented by the paths in the Brieskorn-Pham family given by $a_0=e^{it}, a_1=1$ and $a_0=1,a_1=e^{it}$ respectively.

According to our remark above $\delta _0$ may be identified with elements represented by a loop in the coefficient $a_0=z$ of $x_0^{3d}$ for any sufficiently small perturbation of the Brieskorn-Pham polynomial.

In particular $\delta _0$ identifies with the element of the same name from (3), and therefore can be expressed in the geometric basis $t_i$ of $\pi _1(L_0-{\mathcal {D}}_L)$:

$$\begin{aligned} \delta _0\quad =\quad t_{6d-2} t_{6d-3} \cdots t_2 t_1. \end{aligned}$$

But in $\pi _1({\mathcal {U}}_{d})$ there is another useful expression:

### Lemma 5.7

The element $\delta _0\in \pi _1({\mathcal {U}}_{d})$ is given by

$$\begin{aligned} \delta _0 = t_{2n}t_n\; t_{2n-1} t_{n-1} \;\cdots \; t_{n+2}t_2 \; t_{n+1}t_1 \end{aligned}$$

### Proof

In fact the expression above and in the claim are equivalent under the relations given in the statement of Theorem 2: If in the claim $t_k$ is left of $t_l$ with $k<l$, then $l<n+k$ and therefore $t_kt_l = t_lt_k$. Thus we may order the factors by decreasing index without changing the product and thus arrive at the expression given first. $\square$

The elements $\delta _0,\delta _1$ describe the first map in Proposition 5.5:

### Lemma 5.8

The elements $\delta _0,\delta _1$ commute and

$$\begin{aligned} (\delta _0\delta _1)^6= \delta _0^6\delta _1^6 \end{aligned}$$

is the image in $\pi _1({\mathcal {U}}_{d})$ of a generator of $\pi _1({\mathbb {C}}^*)$.

### Proof

The family $y^3+ a_0x_0^{3d}+a_1x_1^{3d}$ has discriminant given by the normal crossing divisor $\{ a_0a_1=0\}$ and hence the fundamental group of the complement is abelian. Therefore $\delta _0,\delta _1$ commute, since they are geometric generators of the two components.

Since ${\mathbb {C}}^*$ acts with multiplicity 6 on both $a_0,a_1$ the loop of elements of modulus one maps to $(\delta _0\delta _1)^6$. $\square$

### Lemma 5.9

The image in $\pi _1({\mathcal {U}}_{d})$ of a generator of $\pi _1({\text {GL}}_2)$ is given by

$$\begin{aligned} \delta _0^{3d}\quad = \quad \delta _1^{3d}. \end{aligned}$$

### Proof

Both elements $\delta _0,\delta _1$ are represented by the trace of the Brieskorn-Pham point $y^3+x_0^{3d}+x_1^{3d}$ transported by the ${\mathbb {C}}^*$ action on one coefficient, $a_0$ resp. $a_1$. The loops $$({\begin{matrix} e^{it} &{} 0 \\ 0 &{} 1\end{matrix}})$$ and $$({\begin{matrix} 1 &{} 0 \\ 0 &{} e^{it}\end{matrix}})$$ of matrices both represent a generator of $\pi _1({\text {GL}}_2)$. Their action on one variable $x_0$ resp. *x* has the same effect as the ${\mathbb {C}}^*$ action with multiplicity ${3d}$ on the coefficient $a_0$ resp. $a_1$, hence the claim. $\square$

We still have to determine an expression for $\delta _1$ in the given generators. Let us first see, how the two geometric Hefez-Lazzeri bases for positive real $v_0> v_1$ and $v_1>v_0$ compare.

### Lemma 5.10

Consider the Hefez Lazzeri family

$$\begin{aligned} y^3-v_0yx_0^{2d} +x_1^{3d}-v_1x_1x_0^{{3d}-1} +zx_0^{3d}. \end{aligned}$$

Suppose $t_{i,j}$ and $t'_{j',i'}$ form geometric Hefez-Lazzeri bases for positive real $v_0> v_1$ respectively $v_0'= v_1,v_1'=v_0$ of sufficiently distinct magnitude, then there is a path connecting the base points such that the associated isomorphism on fundamental groups is given by

$$\begin{aligned} t_{i,j}\quad \mapsto \quad t_{j,i}'\quad \end{aligned}$$

### Proof

We first convince ourselves that $t_{1,1}=t'_{1,1}$ which follows immediately if we change $v_0,v_1$ continuously in the real line swapping places since the extremal real puncture will keep that property and hence the corresponding geometric element will not be changed.

To move $t_{ij}$ we first proceed along a path $\omega _{ij}$ as in Lemma 3.4 so that it becomes the $t_{11}$ in the new system, then do the same as above and finally employ the corresponding path $\omega '_{ij}$ back again to come to the final position.

The paths thus needed all connect the two base points with $v_0,v_1$ interchanged albeit in different ways. Still all these concatenated paths are homotopic in the complement of the cuspidal bifurcation component. They may be non-homotopic in the complement of the Maxwell bifurcation component, so the induced isomorphisms differ at most by commutators. However, the braid transformations corresponding to the Maxwell component just impose the commutation relations, which are needed to have these commutators being trivial in the fundamental group. $\square$

### Lemma 5.11

The element $\delta _1$ in $\pi _1({\mathcal {U}}_{d})$ can be expressed in the geometric basis $t_1, \dots , t_{2n}$ with $n={3d}-1$:

$$\begin{aligned} \delta _1\quad = \quad t_{n+1} t_1\; t_{n+2} t_2 \,\cdots \, t_{2n}t_{n} \end{aligned}$$

### Proof

We need to switch between the single index notation and the double index notation. Apart from that it suffices by the previous Lemma to show that $\delta _1$ can be expressed in the geometric basis $t'_{ji}$ as

$$\begin{aligned} \delta _1 \quad = \quad t'_{1,2} t'_{1,1} \; t'_{2,2} t'_{2,1}\; \cdots \; t'_{{3d}-1,2} t'_{{3d}-1,1} \end{aligned}$$

We introduce the elements $t^+_j=t'_{j,2} t'_{j,1}$ and observe that they are identified with the elements of the geometric basis for the discriminant complement of the family

$$\begin{aligned} y^3 +x_1^{3d}-v_1x_1x_0^{{3d}-1} +zx_0^{3d}. \end{aligned}$$

along a path, where $v_0$ goes to 0.

Next we consider the result of Zariski. Let ${\mathcal {U}}_{{\mathbf {P}}^1,l}$ as in Theorem 1 denote the discriminant complement associated to the complete linear system of degree *l* on ${\mathbf {P}}^1$. It is the quotient of the subset ${\tilde{{\mathcal {U}}}}_l\in {\mathbb {C}}[x_0,x_1]_l$ of homogeneous polynomials of degree *l* defining *l* distinct points in ${\mathbf {P}}^1$ modulo the diagonal ${\mathbb {C}}^*$ action.

We are going to exploit the natural map

$$\begin{aligned} {\tilde{{\mathcal {U}}}}_{{3d}} \quad \rightarrow \quad {\mathcal {U}}_{d}, \qquad f' \quad \mapsto \quad f=y^3+f' \end{aligned}$$

It identifies the geometric basis of Zariski with the elements $t^+_1,\dots , t^+_{{3d}-1}$ and determines $\delta _0$ and $\delta _1$ as elements of $\pi _1({\tilde{{\mathcal {U}}}}_l)$.

Consider the exact sequence associated to the diagonal ${\mathbb {C}}^*$ action:

$$\begin{aligned} 1\longrightarrow \pi _1({\mathbb {C}}^*)\longrightarrow \pi _1({\tilde{{\mathcal {U}}}}_{3d})\longrightarrow \pi _1({\mathcal {U}}_{{\mathbf {P}}^1,{3d}}) \longrightarrow 1 \end{aligned}$$

Then $\delta _1\delta _0\in \pi _1({\tilde{{\mathcal {U}}}}_{3d})$ is the image of a generator of $\pi _1({\mathbb {C}}^*)$ and we write $\delta _0\,=\, t^+_{{3d}-1}\cdots t^+_1$ using Lemma 5.7. Exploiting Zariski’s result (cf. Theorem 1)

$$\begin{aligned} t^+_1\cdots t^+_{{3d}-1}t^+_{{3d}-1}\cdots t^+_1= 1 \end{aligned}$$

we conclude $\delta _1\,=\, t^+_1\cdots t^+_{{3d}-1}$ as claimed. $\square$

With all ingredients at hand we can finally prove the presentation for the orbifold fundamental group of ${\mathcal {M}}_{d}$.

### Proof of Theorem 4

Proposition 5.5 states, that the presentation is obtained from a presentation of $\pi _1({\mathcal {U}}_{d})$ imposing additional relations from the group action.

That is why most of the presentation is already given in the statement of Theorem 2. In fact, the image of the fundamental group of ${\mathbb {C}}^*\times {\text {GL}}_{2}$ is generated by

$$\begin{aligned} \delta _0^6\delta _1^6 \quad \text { (Lemma 5.7)} \quad \text { and } \qquad \delta _0^{3d}\quad \text { (Lemma 5.8)}. \end{aligned}$$

We express both elements in the generators $t_1,\dots , t_{6d-2}$ using Lemmas 5.7 and 5.11 respectively. Thus we finally arrive at the last line of Theorem 4 giving the two additional relations:

$$\begin{aligned} \quad \mathrm{(v)}\quad \left( t_{n+1} t_1\,\dots \, t_{2n}t_{n} \right) ^6 \big ( t_{2n}t_n\;\dots \; t_{n+1}t_1 \big )^6 =\; 1 \;= \big ( t_{2n}t_n\;\dots \; t_{n+1}t_1 \big )^{3d} \end{aligned}$$

$$\square $$

## Appendix A. An alternative presentation

In this appendix we will transform our presentations purely algebraically. So we get alternative presentations with higher symmetry but more generators. The presentation of Corollary 1 instead is distinguished, since the only relations are braid relations between generators, commutation relations and triviality of two words in all positive generators.

### Definition A.1

Let $G=G_n$ be the finitely presented group with generators

$$\begin{aligned} a_i,\, b_i,\, q_i,\quad 1\le i \le n \end{aligned}$$

and relations given by

$$\begin{aligned} \begin{array}{c} a_i a_j {\mathop {=\!\!=}\limits ^{(a| a)}} a_ja_i, \quad b_ib_j {\mathop {=\!\!=}\limits ^{(b|b)}} b_j b_i, \quad a_i b_j {\mathop {=\!\!=}\limits ^{(a| b)}} b_ja_i, \quad a_ib_{i+1} {\mathop {=\!\!=}\limits ^{(ab+)}} b_{i+1}a_i, \\ a_i a_{i+1} a_i {\mathop {=\!\!=}\limits ^{(aa+a)}} a_{i+1} a_i a_{i+1}, \quad b_i b_{i+1} b_i {\mathop {=\!\!=}\limits ^{(bb+b)}} b_{i+1} b_i b_{i+1} \\ b_i a_{i+1} b_i {\mathop {=\!\!=}\limits ^{(ba+b)}} a_{i+1} b_i a_{i+1}, \quad a_i b_i a_i {\mathop {=\!\!=}\limits ^{(aba)}} b_i a_i b_i, \\ a_ib_ia_{i+1}a_i {\mathop {=\!\!=}\limits ^{(aba+a)}} b_ia_{i+1}a_ib_i, \quad b_{i+1}b_{i}a_{i+1}b_{i+1} {\mathop {=\!\!=}\limits ^{(bb-ab)}} b_{i}a_{i+1}b_{i+1}b_{i}, \\ a_i b_i {\mathop {=\!\!=}\limits ^{(abq)}} b_i q_i. \end{array} \end{aligned}$$

where all indices are in $\{1,\dots ,n\}$ and $|i-j|>1$.

(The decoration on the relations is used later for reference).

### Lemma A.2

The map $t_{1,i}\mapsto b_i, t_{2,i}\mapsto a_i$ defined on the generators of the presentation in Theorem 5 induces an isomorphism of the discriminant knot group of $x^3+y^{n+1}$ onto *G*.

### Proof

The given map induces an identification of generators and relations except for the generators $q_i$ and the relations $a_ib_i=b_iq_i$. Hence they differ only by Tietze transformations, so the claim holds. $\square$

### Proposition A.3

In the group *G* there are additional relations for $|i-j|>1$

6 $$\begin{aligned} a_i q_j = q_j a_i, \quad b_i q_j = q_j b_i, \quad q_i q_j = q_j q_i \end{aligned}$$

and for $1\le i \le n$ ( resp. $1\le i<n$ if $i+1$ occurs):

7 $$\begin{aligned}&\begin{array}{rcl} b_i q_i &{} {\mathop {=\!\!=}\limits ^{(bqa)}} &{} q_i a_i , \\ b_ia_{i+1}a_ib_i &{} {\mathop {=\!\!=}\limits ^{(ba+ab)}} &{} a_{i+1}a_ib_ia_{i+1}, \end{array} \quad \begin{array}{rcl} q_i a_i &{} {\mathop {=\!\!=}\limits ^{(qab)}} &{} a_i b_i, \\ a_{i+1}b_{i+1}b_{i}a_{i+1} &{} {\mathop {=\!\!=}\limits ^{(abb-a)}} &{} b_{i+1}b_{i}a_{i+1}b_{i+1}, \end{array} \end{aligned}$$

8 $$\begin{aligned}&\begin{array}{rcl} q_i a_i q_i &{} {\mathop {=\!\!=}\limits ^{(qaq)}} &{} a_i q_i a_i, \\ q_i b_i q_i &{} {\mathop {=\!\!=}\limits ^{(qbq)}} &{} b_i q_i b_i, \end{array} \quad \begin{array}{rcl} q_i a_{i+1} &{} {\mathop {=\!\!=}\limits ^{(qa+)}} &{} a_{i+1} q_i, \\ b_i q_{i+1} &{} {\mathop {=\!\!=}\limits ^{(bq+)}} &{} q_{i+1} b_i \end{array} \end{aligned}$$

9 $$\begin{aligned}&\begin{array}{rcccl} b_i q_i b_{i+1} b_i &{} {\mathop {=\!\!=}\limits ^{(bqb+b)}} &{} q_i b_{i+1} b_i q_i &{} {\mathop {=\!\!=}\limits ^{(qb+bq)}} &{} b_{i+1} b_i q_i b_{i+1} , \\ q_{i+1} a_{i+1} a_i q_{i+1} &{} {\mathop {=\!\!=}\limits ^{(qaa-q)}} &{} a_{i+1} a_i q_{i+1} a_{i+1} &{} {\mathop {=\!\!=}\limits ^{(aa-qa)}} &{} a_i q_{i+1} a_{i+1} a_i \end{array} \end{aligned}$$

10 $$\begin{aligned}&\begin{array}{rcl} q_i b_{i+1} q_i &{} {\mathop {=\!\!=}\limits ^{(qb+q)}} &{} b_{i+1} q_i b_{i+1} \\ a_i q_{i+1} a_i &{} {\mathop {=\!\!=}\limits ^{(aq+a)}} &{} q_{i+1} a_i q_{i+1} \end{array} \end{aligned}$$

11 $$\begin{aligned}&\begin{array}{rcccl} q_i b_{j+1} q_{j+1} q_j &{} {\mathop {=\!\!=}\limits ^{(q-bqq-)}} &{} b_{j+1} q_{j+1} q_j b_{j+1} &{} {\mathop {=\!\!=}\limits ^{(bqq-b)}} &{} q_{j+1} q_j b_{j+1} q_{j+1} \\ q_j a_j q_{j+1} q_j &{} {\mathop {=\!\!=}\limits ^{(qaq+q)}} &{} a_j q_{j+1} q_j a_j &{} {\mathop {=\!\!=}\limits ^{(aq+qa)}} &{} q_{j+1} q_j a_j q_{j+1} \end{array} \end{aligned}$$

12 $$\begin{aligned}&q_i q_{i+1} q_i \quad {\mathop {=\!\!=}\limits ^{(qq+q)}}\quad q_{i+1} q_i q_{i+1} \end{aligned}$$

### Proof

The first line of relations is immediate from the first and last line of the relations given in the definition. The other relations we address in the given order, and use in each step only transformations established in earlier steps.

$$\begin{aligned} \begin{array}{lc} (7)&\begin{array}[t]{rcccccl} b_j ( b_j q_j) &{} {\mathop {=\!\!=}\limits ^{abq}} &{} b_j (a_j b_j) &{} {\mathop {=\!\!=}\limits ^{aba}} &{} a_j b_j a_j &{} {\mathop {=\!\!=}\limits ^{abq}} &{} b_j (q_j a_j) \end{array} \\ &{} \begin{array}[t]{rcl} ( b_j a_{j+1} a_j b_j ) a_j &{} {\mathop {=\!\!=}\limits ^{aba}} &{} b_j a_{j+1} b_j a_j b_j \\ &{} {\mathop {=\!\!=}\limits ^{ba+b}} &{} a_{j+1} b_j a_{j+1} a_j b_j \\ &{} {\mathop {=\!\!=}\limits ^{aba+a}} &{} (a_{j+1} a_j b_j a_{j+1} ) a_j \end{array} \quad \begin{array}[t]{rcl} b_{j}( a_{j+1} b_{j+1} b_{j} a_{j+1} ) &{} {\mathop {=\!\!=}\limits ^{bb-ab}} &{} b_{j+1} b_{j} a_{j+1} b_{j+1} a_{j+1} \\ &{} {\mathop {=\!\!=}\limits ^{aba}} &{} b_{j+1} b_{j} b_{j+1} a_{j+1} b_{j+1} \\ &{} {\mathop {=\!\!=}\limits ^{bb+b}} &{} b_{j} (b_{j+1} b_{j} a_{j+1} b_{j+1} ) \end{array} \\ (8) &{} \begin{array}[t]{rcl} q_j a_j q_j &{} {\mathop {=\!\!=}\limits ^{qab}} &{} a_j b_j q_j \\ &{} {\mathop {=\!\!=}\limits ^{bqa}} &{} a_j q_j a_j \\ \\ q_j b_j q_j &{} {\mathop {=\!\!=}\limits ^{abq}} &{} q_j a_j b_j \\ &{} {\mathop {=\!\!=}\limits ^{bqa}} &{} b_j q_j b_j \\ \\ \end{array} \quad \begin{array}[t]{rcl} b_j ( q_j a_{j+1}) a_j&{} {\mathop {=\!\!=}\limits ^{abq}} &{} a_j b_j a_{j+1} a_j \\ &{} {\mathop {=\!\!=}\limits ^{aba+a}} &{} b_j a_{j+1} a_j b_j \\ &{} {\mathop {=\!\!=}\limits ^{qab}} &{} b_j ( a_{j+1} q_j ) a_j \\ b_{j+1} ( b_j q_{j+1}) a_{j+1} &{} {\mathop {=\!\!=}\limits ^{qab}} &{} b_{j+1} b_j a_{j+1} b_{j+1} \\ &{} {\mathop {=\!\!=}\limits ^{abb-a}} &{} a_{j+1} b_{j+1} b_j a_{j+1} \\ &{} {\mathop {=\!\!=}\limits ^{abq}} &{} b_{j+1} ( q_{j+1} b_j) a_{j+1} \end{array} \\ (9) &{} \begin{array}[t]{rcl} b_j q_j b_{j+1} b_j &{} {\mathop {=\!\!=}\limits ^{bqa}} &{} q_j a_j b_{j+1} b_j \\ &{} {\mathop {=\!\!=}\limits ^{ab+}} &{} q_j b_{j+1} a_j b_j \\ &{} {\mathop {=\!\!=}\limits ^{abq}} &{} q_j b_{j+1} b_j q_j \\ \\ \end{array} \quad \begin{array}[t]{rcl} b_j q_j b_{j+1} b_j &{} {\mathop {=\!\!=}\limits ^{abq}} &{} a_j b_j b_{j+1} b_j \\ &{} {\mathop {=\!\!=}\limits ^{bbb}} &{} a_j b_{j+1} b_j b_{j+1} \\ &{} {\mathop {=\!\!=}\limits ^{ab+}} &{} b_{j+1} a_j b_j b_{j+1} \\ &{} {\mathop {=\!\!=}\limits ^{abq}} &{} b_{j+1} b_j q_j b_{j+1} \end{array} \\ &{} \begin{array}{rcl} q_{j+1} a_{j+1} a_j q_{j+1} &{} {\mathop {=\!\!=}\limits ^{qab}} &{} a_{j+1} b_{j+1} a_j q_{j+1} \\ &{} {\mathop {=\!\!=}\limits ^{ab+}} &{} a_{j+1} a_j b_{j+1} q_{j+1} \\ &{} {\mathop {=\!\!=}\limits ^{bqa}} &{} a_{j+1} a_j q_{j+1} a_{j+1} \\ \\ \end{array} \quad \begin{array}{rcl} a_j q_{j+1} a_{j+1} a_j &{} {\mathop {=\!\!=}\limits ^{qab}} &{} a_{j} a_{j+1} b_{j+1} a_j \\ &{} {\mathop {=\!\!=}\limits ^{ab+}} &{} a_j a_{j+1} a_j b_{j+1} \\ &{} {\mathop {=\!\!=}\limits ^{aaa}} &{} a_{j+1} a_j a_{j+1} b_{j+1} \\ &{} {\mathop {=\!\!=}\limits ^{qab}} &{} a_{j+1} a_j q_{j+1} a_{j+1} \end{array} \\ \end{array} \end{aligned}$$

$$\begin{aligned} \begin{array}{lc} (10) &{} \begin{array}[t]{rcl} ( q_j b_{j+1} q_j )b_j q_j &{} {\mathop {=\!\!=}\limits ^{qbq}} &{} q_j b_{j+1} b_j q_j b_j \\ &{} {\mathop {=\!\!=}\limits ^{qb+bq}} &{} b_{j+1} b_j q_j b_{j+1} b_j \\ &{} {\mathop {=\!\!=}\limits ^{bqb+b}} &{} (b_{j+1} q_j b_{j+1} ) b_j q_j \end{array} \quad \begin{array}[t]{rcl} ( a_j q_{j+1} a_j )a_{j+1} a_j &{} {\mathop {=\!\!=}\limits ^{aa+a}} &{} a_j q_{j+1} a_{j+1} a_j a_{j+1} \\ &{} {\mathop {=\!\!=}\limits ^{qaa-q}} &{} q_{j+1} a_{j+1} a_j q_{j+1} a_{j+1} \\ &{} {\mathop {=\!\!=}\limits ^{aa-qa}} &{} ( q_{j+1} a_j q_{j+1} ) a_{j+1} a_j \end{array} \\ (11) &{} \begin{array}[t]{rcl} q_j b_{j+1} q_{j+1} q_{j} &{} {\mathop {=\!\!=}\limits ^{abq}} &{} q_{j+1} a_j b_j q_{j+1} \\ &{} {\mathop {=\!\!=}\limits ^{bq+}} &{} q_{j+1} a_j q_{j+1} b_j \\ &{} {\mathop {=\!\!=}\limits ^{aq+a}} &{} a_j q_{j+1} a_j b_j \\ &{} {\mathop {=\!\!=}\limits ^{abq}} &{} b_{j+1} q_{j+1} q_j b_{j+1} \\ b_{j+1} q_{j+1} q_j b_{j+1} &{} {\mathop {=\!\!=}\limits ^{bqa}} &{} q_{j+1} a_{j+1} q_j b_{j+1} \\ &{} {\mathop {=\!\!=}\limits ^{qa+}} &{} q_{j+1} q_j a_{j+1} b_{j+1} \\ &{} {\mathop {=\!\!=}\limits ^{abq}} &{} q_{j+1} q_j b_{j+1} q_{j+1} \end{array} \quad \begin{array}[t]{rcl} a_j q_{j+1} q_j a_j &{} {\mathop {=\!\!=}\limits ^{abq}} &{} a_j q_{j+1} a_j b_j \\ &{} {\mathop {=\!\!=}\limits ^{aq+a}} &{} q_{j+1} a_j q_{j+1} b_j \\ &{} {\mathop {=\!\!=}\limits ^{bq+}} &{} q_{j+1} a_j b_j q_{j+1} \\ &{} {\mathop {=\!\!=}\limits ^{qab}} &{} q_{j+1} q_j a_j q_{j+1} \\ q_j a_j q_{j+1} q_j &{} {\mathop {=\!\!=}\limits ^{qab}} &{} a_j b_j q_{j+1} q_j \\ &{} {\mathop {=\!\!=}\limits ^{bq+}} &{} a_j q_{j+1} b_j q_j \\ &{} {\mathop {=\!\!=}\limits ^{bqa}} &{} a_j q_{j+1} q_j a_j \end{array} \\ (12) &{} \begin{array}[t]{rcl} ( q_j q_{j+1} q_j ) b_{j+1} q_{j+1} &{} {\mathop {=\!\!=}\limits ^{bqq-b}} &{} q_j b_{j+1} q_{j+1} q_j b_{j+1} \\ &{} {\mathop {=\!\!=}\limits ^{qq-bq}} &{} q_{j+1} q_j b_{j+1} q_{j+1} b_{j+1} \\ &{} {\mathop {=\!\!=}\limits ^{qbq}} &{} (q_{j+1} q_j q_{j+1} ) b_{j+1} q_{j+1} \\ \end{array} \end{array} \end{aligned}$$

### Definition A.4

Let $S=S_n$ be the finitely presented group with generators

$$\begin{aligned} \sigma _i,\quad 1\le i \le 2n \end{aligned}$$

and relations given by

$$\begin{aligned}&\sigma _i\sigma _j\sigma _i {\mathop {=\!\!=}\limits ^{(brai)}} \sigma _j\sigma _i\sigma _j&\text {if }\ |i-j|\le 2\\&\sigma _i\sigma _j {\mathop {=\!\!=}\limits ^{(com)}} \sigma _j\sigma _i&\text {if }\ |i-j|>2\\&\sigma _{i+1}\sigma _{i-1}\sigma _{i} \sigma _{i+1} {\mathop {=\!\!=}\limits ^{(cyc)}} \sigma _{i-1}\sigma _{i}\sigma _{i+1}\sigma _{i-1}&\text {if}\ 1<i<2n \end{aligned}$$

and let $S^*=S^*_n$ be the finitely presented group obtained from *S* by the Tietze transformation adding the additional generators and relations

$$\begin{aligned} \tau _{2i},\quad \tau _{2i}\sigma _{2i-1} {\mathop {=\!\!=}\limits ^{(\tau \sigma '\sigma )}} \sigma _{2i-1}\sigma _{2i}, \quad 1\le i \le n \end{aligned}$$

### Proposition A.5

In the group $S^*$ the following relations hold:

13 $$\begin{aligned}&\begin{array}{rcl} \sigma _{2i-1}\sigma _{2i} \quad {\mathop {=\!\!=}\limits ^{(\sigma '\sigma \tau )}} &{} \sigma _{2i} \tau _{2i} &{} {\mathop {=\!\!=}\limits ^{(\sigma \tau \sigma ')}} \quad \tau _{2i}\sigma _{2i-1} , \\ \sigma _{i-1}\sigma _{i}\sigma _{i+1}\sigma _{i-1} &{} {\mathop {=\!\!=}\limits ^{(cyc')}} &{} \sigma _{i}\sigma _{i+1}\sigma _{i-1}\sigma _{i}, \end{array} \nonumber \\&\begin{array}{rcl} \tau _{2i} \sigma _{2i} \tau _{2i} &{} {\mathop {=\!\!=}\limits ^{(13.1)}} &{} \sigma _{2i} \tau _{2i} \sigma _{2i}, \\ \tau _{2i} \sigma _{2i-1} \tau _{2i} &{} {\mathop {=\!\!=}\limits ^{(13.2)}} &{} \sigma _{2i-1}\tau _{2i} \sigma _{2i-1}, \end{array} \quad \begin{array}{rcl} \tau _{2i} \sigma _{2i+1} &{} {\mathop {=\!\!=}\limits ^{(13.3)}} &{} \sigma _{2i+1} \tau _{2i}, \\ \sigma _{2i} \tau _{2i+2} &{} {\mathop {=\!\!=}\limits ^{(13.4)}} &{} \tau _{2i+2} \sigma _{2i} \end{array} \end{aligned}$$

14 $$\begin{aligned}&\begin{array}{rcccl} \sigma _{2i} \tau _{2i} \sigma _{2i+2} \sigma _{2i} &{} {\mathop {=\!\!=}\limits ^{(14.1)}} &{} \tau _{2i} \sigma _{2i+2} \sigma _{2i} \tau _{2i} &{} {\mathop {=\!\!=}\limits ^{(14.2)}} &{} \sigma _{2i+2} \sigma _{2i} \tau _{2i} \sigma _{2i+2}, \\ \tau _{2i+2} \sigma _{2i+1} \sigma _{2i-1} \tau _{2i+2} &{} {\mathop {=\!\!=}\limits ^{(14.3)}} &{} \sigma _{2i+1} \sigma _{2i-1} \tau _{2i+2} \sigma _{2i+1} &{} {\mathop {=\!\!=}\limits ^{(14.4)}} &{} \sigma _{2i-1} \tau _{2i+2} \sigma _{2i+1} \sigma _{2i-1} \end{array} \end{aligned}$$

15 $$\begin{aligned}&\begin{array}{rcl} \tau _{2i} \sigma _{2i+2} \tau _{2i} &{} {\mathop {=\!\!=}\limits ^{(15.1)}} &{} \sigma _{2i+2} \tau _{2i} \sigma _{2i+2} \\ \sigma _{2i-1} \tau _{2i+2} \sigma _{2i-1} &{} {\mathop {=\!\!=}\limits ^{(15.2)}} &{} \tau _{2i+2} \sigma _{2i-1} \tau _{2i+2} \end{array} \end{aligned}$$

16 $$\begin{aligned}&\begin{array}{rcccl} \tau _{2i} \sigma _{2i+2} \tau _{2i+2} \tau _{2i} &{} {\mathop {=\!\!=}\limits ^{(16.1)}} &{} \sigma _{2i+2} \tau _{2i+2} q_j \sigma _{2i+2} &{} {\mathop {=\!\!=}\limits ^{(16.2)}} &{} \tau _{2i+2} \tau _{2i} \sigma _{2i+2} \tau _{2i+2} \\ \tau _{2i} \sigma _{2i-1} \tau _{2i+2} \tau _{2i} &{} {\mathop {=\!\!=}\limits ^{(16.3)}} &{} \sigma _{2i-1} \tau _{2i+2} \tau _{2i} \sigma _{2i-1} &{} {\mathop {=\!\!=}\limits ^{(16.4)}} &{} \tau _{2i+2} \tau _{2i} \sigma _{2i-1} \tau _{2i+2} \end{array} \end{aligned}$$

17 $$\begin{aligned}&\tau _{2i} \tau _{2i+2} \tau _{2i} {\mathop {=\!\!=}\limits ^{(17.1)}}\tau _{2i+2} \tau _{2i} \tau _{2i+2} \end{aligned}$$

### Proof

The claims are established in the same way as before:

$$\begin{aligned} \begin{array}{lc} (6)&\begin{array}[t]{rcccccl} ( \tau _{2i} \sigma _{2i-1} ) \sigma _{2i-1} &{} {\mathop {=\!\!=}\limits ^{\tau \sigma '\sigma }} &{} \sigma _{2i-1} \sigma _{2i} \sigma _{2i-1} &{} {\mathop {=\!\!=}\limits ^{brai}} &{} \sigma _{2i} \sigma _{2i-1} \sigma _{2i} &{} {\mathop {=\!\!=}\limits ^{\tau \sigma '\sigma }} &{} (\sigma _{2i} \tau _{2i} ) \sigma _{2i-1} \end{array} \\ &{} \begin{array}[t]{rcl} ( \sigma _{i-1} \sigma _{i} \sigma _{i+1} \sigma _{i-1} )\sigma _{i+1} &{} {\mathop {=\!\!=}\limits ^{brai}} &{} \sigma _{i-1} \sigma _{i} \sigma _{i-1} \sigma _{i+1} \sigma _{i-1} \\ &{} {\mathop {=\!\!=}\limits ^{brai}} &{} \sigma _{i} \sigma _{i-1} \sigma _{i} \sigma _{i+1} \sigma _{i-1} \\ &{} {\mathop {=\!\!=}\limits ^{cyc}} &{} ( \sigma _{i} \sigma _{i+1} \sigma _{i-1} \sigma _{i} ) \sigma _{i+1} \end{array} \\ (7) &{} \begin{array}[t]{rcl} \tau _{2i} \sigma _{2i} \tau _{2i} &{} {\mathop {=\!\!=}\limits ^{\sigma '\sigma \tau }} &{} \tau _{2i} \sigma _{2i-1} \sigma _{2i} \\ &{} {\mathop {=\!\!=}\limits ^{\sigma \tau \sigma '}} &{} \sigma _{2i} \tau _{2i} \sigma _{2i} \\ \tau _{2i} \sigma _{2i-1} \tau _{2i} &{} {\mathop {=\!\!=}\limits ^{\tau \sigma '\sigma }} &{} \tau _{2i} \sigma _{2i} \sigma _{2i-1} \\ &{} {\mathop {=\!\!=}\limits ^{\tau \sigma '\sigma }} &{} \sigma _{2i-1} \tau _{2i} \sigma _{2i-1} \\ \end{array} \quad \begin{array}[t]{rcl} \sigma _{2i} ( \tau _{2i} \sigma _{2i+1}) \sigma _{2i-1}&{} {\mathop {=\!\!=}\limits ^{\sigma '\sigma \tau }} &{} \sigma _{2i-1} \sigma _{2i} \sigma _{2i+1} \sigma _{2i-1} \\ &{} {\mathop {=\!\!=}\limits ^{cyc}} &{} \sigma _{2i} \sigma _{2i+1} \sigma _{2i-1} \sigma _{2i} \\ &{} {\mathop {=\!\!=}\limits ^{\tau \sigma '\sigma }} &{} \sigma _{2i} ( \sigma _{2i+1} \tau _{2i} ) \sigma _{2i-1} \\ \sigma _{2i+2} ( \sigma _{2i} \tau _{2i+2}) \sigma _{2i+1} &{} {\mathop {=\!\!=}\limits ^{\tau \sigma '\sigma }} &{} \sigma _{2i+2} \sigma _{2i} \sigma _{2i+1} \sigma _{2i+2} \\ &{} {\mathop {=\!\!=}\limits ^{cyc}} &{} \sigma _{2i+1} \sigma _{2i+2} \sigma _{2i} \sigma _{2i+1} \\ &{} {\mathop {=\!\!=}\limits ^{\sigma '\sigma \tau }} &{} \sigma _{2i+2} ( \tau _{2i+2} \sigma _{2i}) \sigma _{2i+1} \end{array} \end{array} \end{aligned}$$

$$\begin{aligned} \begin{array}{lc} (9) &{} \begin{array}[t]{rcl} \sigma _{2i} \tau _{2i} \sigma _{2i+2} \sigma _{2i} &{} {\mathop {=\!\!=}\limits ^{\sigma \tau \sigma '}} &{} \tau _{2i} \sigma _{2i-1} \sigma _{2i+2} \sigma _{2i} \\ &{} {\mathop {=\!\!=}\limits ^{com}} &{} \tau _{2i} \sigma _{2i+2} \sigma _{2i-1} \sigma _{2i} \\ &{} {\mathop {=\!\!=}\limits ^{\sigma '\sigma \tau }} &{} \tau _{2i} \sigma _{2i+2} \sigma _{2i} \tau _{2i} \\ \end{array} \quad \begin{array}[t]{rcl} \tau _{2i+2} \sigma _{2i+1} \sigma _{2i-1} \tau _{2i+2} &{} {\mathop {=\!\!=}\limits ^{\tau \sigma '\sigma }} &{} \sigma _{2i+1} \sigma _{2i+2} \sigma _{2i-1} \tau _{2i+2} \\ &{} {\mathop {=\!\!=}\limits ^{com}} &{} \sigma _{2i+1} \sigma _{2i-1} \sigma _{2i+2} \tau _{2i+2} \\ &{} {\mathop {=\!\!=}\limits ^{\sigma \tau \sigma '}} &{} \sigma _{2i+1} \sigma _{2i-1} \tau _{2i+2} \sigma _{2i+1} \\ \end{array} \\ &{} \begin{array}[t]{rcl} \sigma _{2i} \tau _{2i} \sigma _{2i+2} \sigma _{2i} &{} {\mathop {=\!\!=}\limits ^{\sigma '\sigma \tau }} &{} \sigma _{2i-1} \sigma _{2i} \sigma _{2i+2} \sigma _{2i} \\ &{} {\mathop {=\!\!=}\limits ^{brai}} &{} \sigma _{2i-1} \sigma _{2i+2} \sigma _{2i} \sigma _{2i+2} \\ &{} {\mathop {=\!\!=}\limits ^{com}} &{} \sigma _{2i+2} \sigma _{2i-1} \sigma _{2i} \sigma _{2i+2} \\ &{} {\mathop {=\!\!=}\limits ^{\sigma '\sigma \tau }} &{} \sigma _{2i+2} \sigma _{2i} \tau _{2i} \sigma _{2i+2} \end{array} \quad \begin{array}[t]{rcl} \sigma _{2i-1} \tau _{2i+2} \sigma _{2i+1} \sigma _{2i-1} &{} {\mathop {=\!\!=}\limits ^{\tau \sigma '\sigma }} &{} a_{j} \sigma _{2i+1} \sigma _{2i+2} \sigma _{2i-1} \\ &{} {\mathop {=\!\!=}\limits ^{com}} &{} \sigma _{2i-1} \sigma _{2i+1} \sigma _{2i-1} \sigma _{2i+2} \\ &{} {\mathop {=\!\!=}\limits ^{brai}} &{} \sigma _{2i+1} \sigma _{2i-1} \sigma _{2i+1} \sigma _{2i+2} \\ &{} {\mathop {=\!\!=}\limits ^{\tau \sigma '\sigma }} &{} \sigma _{2i+1} \sigma _{2i-1} \tau _{2i+2} \sigma _{2i+1} \end{array} \\ (10) &{} \begin{array}[t]{rcl} ( \tau _{2i} \sigma _{2i+2} \tau _{2i} )\sigma _{2i} \tau _{2i} &{} {\mathop {=\!\!=}\limits ^{13.1}} &{} \tau _{2i} \sigma _{2i+2} \sigma _{2i} \tau _{2i} \sigma _{2i} \\ &{} {\mathop {=\!\!=}\limits ^{14.2}} &{} \sigma _{2i+2} \sigma _{2i} \tau _{2i} \sigma _{2i+2} \sigma _{2i} \\ &{} {\mathop {=\!\!=}\limits ^{14.1}} &{} (\sigma _{2i+2} \tau _{2i} \sigma _{2i+2} ) \sigma _{2i} \tau _{2i} \end{array} \\ &{} \begin{array}[t]{rcl} ( \sigma _{2i-1} \tau _{2i+2} \sigma _{2i-1} )\sigma _{2i+1} \sigma _{2i-1} &{} {\mathop {=\!\!=}\limits ^{brai}} &{} \sigma _{2i-1} \tau _{2i+2} \sigma _{2i+1} \sigma _{2i-1} \sigma _{2i+1} \\ &{} {\mathop {=\!\!=}\limits ^{14.3/4}} &{} \tau _{2i+2} \sigma _{2i+1} \sigma _{2i-1} \tau _{2i+2} \sigma _{2i+1} \\ &{} {\mathop {=\!\!=}\limits ^{14.4}} &{} ( \tau _{2i+2} \sigma _{2i-1} \tau _{2i+2} ) \sigma _{2i+1} \sigma _{2i-1} \end{array} \\ (11) &{} \begin{array}[t]{rcl} \tau _{2i} \sigma _{2i+2} \tau _{2i+2} q_{j} &{} {\mathop {=\!\!=}\limits ^{\sigma '\sigma \tau }} &{} \tau _{2i+2} \sigma _{2i-1} \sigma _{2i} \tau _{2i+2} \\ &{} {\mathop {=\!\!=}\limits ^{13.4}} &{} \tau _{2i+2} \sigma _{2i-1} \tau _{2i+2} \sigma _{2i} \\ &{} {\mathop {=\!\!=}\limits ^{15.2}} &{} \sigma _{2i-1} \tau _{2i+2} \sigma _{2i-1} \sigma _{2i} \\ &{} {\mathop {=\!\!=}\limits ^{\sigma '\sigma \tau }} &{} \sigma _{2i+2} \tau _{2i+2} \tau _{2i} \sigma _{2i+2} \\ \sigma _{2i+2} \tau _{2i+2} \tau _{2i} \sigma _{2i+2} &{} {\mathop {=\!\!=}\limits ^{\sigma \tau \sigma '}} &{} \tau _{2i+2} \sigma _{2i+1} \tau _{2i} \sigma _{2i+2} \\ &{} {\mathop {=\!\!=}\limits ^{13.3}} &{} \tau _{2i+2} \tau _{2i} \sigma _{2i+1} \sigma _{2i+2} \\ &{} {\mathop {=\!\!=}\limits ^{\sigma '\sigma \tau }} &{} \tau _{2i+2} \tau _{2i} \sigma _{2i+2} \tau _{2i+2} \end{array} \quad \begin{array}[t]{rcl} \sigma _{2i-1} \tau _{2i+2} \tau _{2i} \sigma _{2i-1} &{} {\mathop {=\!\!=}\limits ^{\sigma '\sigma \tau }} &{} \sigma _{2i-1} \tau _{2i+2} \sigma _{2i-1} \sigma _{2i} \\ &{} {\mathop {=\!\!=}\limits ^{15.2}} &{} \tau _{2i+2} \sigma _{2i-1} \tau _{2i+2} \sigma _{2i} \\ &{} {\mathop {=\!\!=}\limits ^{13.4}} &{} \tau _{2i+2} \sigma _{2i-1} \sigma _{2i} \tau _{2i+2} \\ &{} {\mathop {=\!\!=}\limits ^{\tau \sigma '\sigma }} &{} \tau _{2i+2} \tau _{2i} \sigma _{2i-1} \tau _{2i+2} \\ \tau _{2i} \sigma _{2i-1} \tau _{2i+2} \tau _{2i} &{} {\mathop {=\!\!=}\limits ^{\tau \sigma '\sigma }} &{} \sigma _{2i-1} \sigma _{2i} \tau _{2i+2} \tau _{2i} \\ &{} {\mathop {=\!\!=}\limits ^{13.4}} &{} \sigma _{2i-1} \tau _{2i+2} \sigma _{2i} \tau _{2i} \\ &{} {\mathop {=\!\!=}\limits ^{\sigma \tau \sigma '}} &{} \sigma _{2i-1} \tau _{2i+2} \tau _{2i} \sigma _{2i-1} \end{array} \\ (12) &{} \begin{array}[t]{rcl} ( \tau _{2i} \tau _{2i+2} \tau _{2i} ) \sigma _{2i+2} \tau _{2i+2} &{} {\mathop {=\!\!=}\limits ^{16.2}} &{} \tau _{2i} \sigma _{2i+2} \tau _{2i+2} \tau _{2i} \sigma _{2i+2} \\ &{} {\mathop {=\!\!=}\limits ^{16.1/2}} &{} \tau _{2i+2} \tau _{2i} \sigma _{2i+2} \tau _{2i+2} \sigma _{2i+2} \\ &{} {\mathop {=\!\!=}\limits ^{13.1}} &{} (\tau _{2i+2} \tau _{2i} \tau _{2i+2} ) \sigma _{2i+2} \tau _{2i+2} \\ \end{array} \end{array} \end{aligned}$$

### Proposition A.6

The group *G* is presented in terms of the oriented graph ( the second vertex of an edge is marked by a point close by) with generators given by the vertices *v* of the graph and relations

$$\begin{aligned} \left\langle v\in V \quad \Bigg | \begin{array}{rccl} &{} &{} {v_1} {v_2}={v_2} {v_1}, &{}(v_1,v_2)\not \in E,\\ &{}&{} {v_1} {v_2} {v_1}={v_2} {v_1} {v_2}, &{} (v_1,v_2)\in E,\\ &{}&{} {v_1} {v_3} {v_2} {v_1}={v_2} {v_1} {v_3} {v_2}, &{} (v_1,v_2),(v_1,v_3),(v_2,v_3)\in E \\ &{}&{} {v_1} {v_2}={v_2} {v_3}, &{} (v_1,v_2),(v_2,v_3),(v_3,v_1)\in E \end{array} \right\rangle \end{aligned}$$

### Proof

It suffices to check that the relations of the claim are those of the definition of *G* together with all additional ones from Proposition A.3, if we identify the generators on the top line with the elements $a_1$ to $a_n$, the elements on the bottom line with the elements $b_1$ to $b_n$ and the elements on the line in the back with the elements $q_1$ to $q_n$, from left to right in each case. $\square$

### Proposition A.7

The group *S* has the same presentation as the group *G* under the map induced by the bijection on generators

$$\begin{aligned} \begin{array}{c} \sigma _{2i-1} \mapsto \left\{ \begin{array}{lll} a_{n+1-i} &{} i \equiv _3 n \\ b_{n+1-i} &{} i \equiv _3 n+1 \\ q_{n+1-i} &{} i \equiv _3 n-1 \\ \end{array} \right. \qquad \sigma _{2i} \mapsto \left\{ \begin{array}{lll} a_{n+1-i} &{} i \equiv _3 n-1 \\ b_{n+1-i} &{} i \equiv _3 n \\ q_{n+1-i} &{} i \equiv _3 n+1 \\ \end{array} \right. \\ \tau _{2i} \mapsto \left\{ \begin{array}{lll} a_{n+1-i} &{} i \equiv _3 n+1 \\ b_{n+1-i} &{} i \equiv _3 n-1 \\ q_{n+1-i} &{} i \equiv _3 n \\ \end{array} \right. \end{array} \end{aligned}$$

### Proof

The given map identifies the group $S^*$ with the group of the last proposition. In fact the relations from the definition of $S^*$ and the relations from Proposition A.5 correspond under this map to the full set of relations in the last proposition. $\square$

### Lemma A.8

$$\begin{aligned} a_i \delta = \delta q_{i+1}, \quad b_i \delta = \delta a_{i+1}, \quad q_i \delta = \delta b_{i+1} \end{aligned}$$

### Proof

We prove the second relation using the expression $\delta = a_n b_n \cdots a_1 b_1$:

$$\begin{aligned} \begin{array}{rclcl} b_i\delta &{} = &{} a_n b_n \cdots a_{i+2} b_{i+3} \quad b_i\quad a_{i+1} b_{i+1} a_i b_i \quad a_{i-1} b_{i-1} \cdots a_1 b_1 \\ &{} = &{} a_n b_n \cdots a_{i+2} b_{i+3} \quad b_i\quad q_{i+1} a_{i+1} a_i b_i \quad a_{i-1} b_{i-1} \cdots a_1 b_1 \\ &{} = &{} a_n b_n \cdots a_{i+2} b_{i+3} \quad q_{i+1} \quad b_i a_{i+1} a_i b_i \quad a_{i-1} b_{i-1} \cdots a_1 b_1 \\ &{} = &{} a_n b_n \cdots a_{i+2} b_{i+3} \quad q_{i+1} \quad a_{i+1} a_i b_i a_{i+1} \quad a_{i-1} b_{i-1} \cdots a_1 b_1 \\ &{} = &{} a_n b_n \cdots a_{i+2} b_{i+3} \quad q_{i+1} a_{i+1} \quad a_i b_i \;\, a_{i+1} \;\, a_{i-1} b_{i-1} \cdots a_1 b_1 \\ &{} = &{} a_n b_n \cdots a_{i+2} b_{i+3} \quad a_{i+1} b_{i+1} \quad a_i b_ia_{i-1} b_{i-1} \cdots a_1 b_1\quad a_{i+1} &{} = &{} \delta a_{i+1} \end{array} \end{aligned}$$

The other two then follow in the same way since $\delta = b_nq_n \cdots b_1 q_1 =q_n a_n \cdots q_1 a_1$. $\square$

### Proof of Corollary 1

Under the isomorphisms of Lemma A.2 and Proposition A.7 respectively we get

$$\begin{aligned} t_{n+k} t_{k} \quad = \quad a_k b_k = b_kq_k = q_k a_k \quad = \quad \sigma _{2n+2-2k} \sigma _{2n+1-2k} \end{aligned}$$

Hence we can identify immediately

$$\begin{aligned} t_{2n}t_n\; \cdots \; t_{n+1}t_1 = \sigma _1\sigma _2 \cdots \sigma _{2n}, \quad t_{n+1}t_1\; \cdots \; t_{2n}t_{n} = \sigma _{2n-1}\sigma _{2n}\; \sigma _{2n-3}\sigma _{2n-2} \; \cdots \; \sigma _1\sigma _2. \end{aligned}$$

It remains to prove the equivalence of the last two relations with the relations *iv*):

With $\delta = t_{2n}t_n\; \cdots \; t_{n+1}t_1$ again and *t* either $t_1$ or $t_{n+1}$ we can transform

$$\begin{aligned} \begin{array}{crcl} &{} (t^{^{-1}}\delta )^n &{} = &{} (\delta t^{^{-1}})^n \\ \iff &{} (\delta ^{^{-1}}t)^n &{} = &{} (t \delta ^{^{-1}})^n \\ \iff &{} \delta ^{^{-1}}\; t\; \delta ^{^{-1}}\! t\delta \; \delta ^{-2} t\delta ^2\;\cdots \; \delta ^{-(n-1)}t\delta ^{(n-1)} &{} = &{} t\; \delta ^{^{-1}}\! t\delta \; \delta ^{-2} t\delta ^2\;\cdots \; \delta ^{-(n-1)}t\delta ^{(n-1)}\; \delta ^{^{-1}}\end{array} \end{aligned}$$

Using the previous lemma we get:

$$\begin{aligned} t_1\; \delta ^{^{-1}}\! t_1\delta \; \delta ^{-2} t_1\delta ^2\;\cdot _1s \; \delta ^{-(n-1)}t_1\delta ^{(n-1)}= & {} b_1a_2q_3b_4\cdots \\= & {} \sigma _{2n}\sigma _{2n-2}\sigma _{2n-4}\sigma _{2n-6} \cdots \sigma _4\sigma _2 \\ t_{n+1}\; \delta ^{^{-1}}\! t_{n+1}\delta \; \delta ^{-2} t_{n+1}\delta ^2\;\cdots \; \delta ^{-(n-1)}t_{n+1}\delta ^{(n-1)}= & {} a_1q_2b_3a_4\cdots \\= & {} \sigma _{2n-1} \sigma _{2n-3} \cdots \sigma _3\sigma _1 \end{aligned}$$

$$\square $$
